# A combination of stabilizing selection and random walk are associated with phylogenetic signal in hard pines

**DOI:** 10.1093/aob/mcaf147

**Published:** 2025-07-16

**Authors:** Jorge Cruz-Nicolás, David S Gernandt

**Affiliations:** Departamento de Botánica, Instituto de Biología, Universidad Nacional Autónoma de México, Ciudad de México 04510, México; Instituto de Geografía, Universidad Nacional Autónoma de México, Circuito de la Universidad Nacional Autónoma de México, Circuito de la Investigación Científica s/n, Ciudad de México 04510, México; Departamento de Botánica, Instituto de Biología, Universidad Nacional Autónoma de México, Ciudad de México 04510, México

**Keywords:** Climatic niche, ellipsoids, Ornstein–Uhlenbeck, niche overlap, non-adaptive, phylogenetic niche conservatism, radiation

## Abstract

**Background and Aims:**

Although phylogenetic niche conservatism is widely accepted, in many cases the cause of this conservatism is unclear. The presence of phylogenetic signal could extend to morphological and anatomical characters, chemical soil properties and DNA content. However, as sessile organisms, especially those that migrated toward drier or tropical conditions, are subject to environmental heterogeneity, phylogenetic signal in these characters could be explained by a combination of different evolutionary forces.

**Methods:**

We assessed the phylogenetic signal in DNA content, chemical soil properties, climate and morphoanatomical characteristics, tested different evolutionary models and performed an ancestral state reconstruction in 30 species of hard pines geographically distributed in North America, Mexico, Central America and the Caribbean. To reinforce our hypothesis of phylogenetic niche conservatism, we applied a niche similarity test among different species pairs, performed ecological niche modelling and projected these models in geographical space.

**Key Results:**

We found strong phylogenetic signals in the characters evaluated, indicating a retention of characteristics throughout the evolutionary history of these pines. The best models to explain these phylogenetic signals were Ornstein–Uhlenbeck, Brownian Motion and Early Burst, indicating the action of stabilizing selection, with an input of random walk. The detection of niche overlap supported our hypothesis of phylogenetic niche conservatism; however, we found more similarity than expected in more phylogenetically distant species.

**Conclusions:**

Overall, we detected strong phylogenetic signal, and our results supported the hypothesis of phylogenetic niche conservatism, but there was more similarity in some species that have evolved under similar selective pressures independent of phylogenetic relationships. No single evolutionary model fully explains trait divergence; depending on the specific trait, divergence could be explained primarily by stabilizing selection or random walk in these hard pines.

## Graphical Abstract

**Graphical Abstract:**
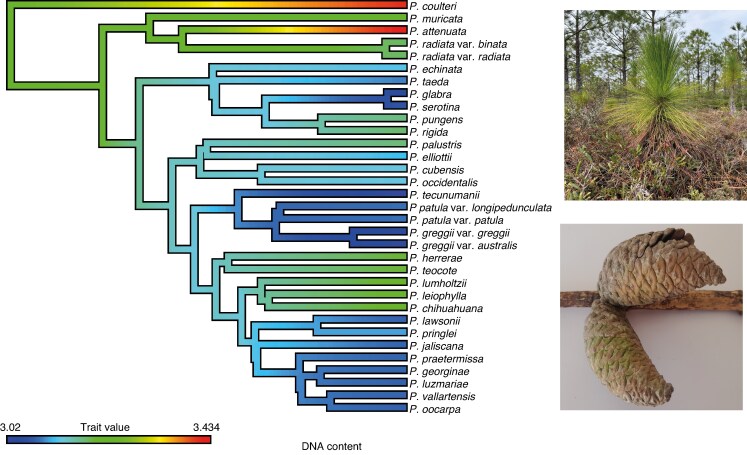

## INTRODUCTION

The divergence of characters over evolutionary history in closely related species, especially in those that have radiated recently, has been a subject of interest in ecology and evolution. On a macroevolutionary scale there is a tendency toward the retention of characters related to climatic tolerance, which is interpreted as phylogenetic niche conservatism (Wiens and Graham, 2005; Wiens *et al*., 2010). This concept is extended to morphological, physiological or functional characteristics (Losos, 2008). Conservatism is linked to shared history among closely related species and could be the product of non-independence in the variation of characters, which is referred to as phylogenetic signal (Felsenstein, 1985; Revell *et al*., 2008; Beaulieu *et al*., 2012).

Evidence of phylogenetic niche conservatism in different organisms is often attributed to Brownian Motion, i.e. ‘random walk’ (Harvey and Pagel, 1991; Crisp and Cook, 2012). However, phylogenetic signal might be the result of other processes, and in these cases Brownian Motion may not be an appropriate model (Revell *et al*., 2008; Münkemüller *et al*., 2012), especially where the variance does not increase with time, possibly because stabilizing selection maintains traits around an optimal value (Hansen, 1997; Butler and King, 2004). This could be relevant in species that have radiated along latitudinal gradients, such as the hard pines that presumably migrated from North America to more southern latitudes, or when these pines have been subjected to greater environmental heterogeneity. In either case, we could expect different alternatives to Brownian Motion (He *et al*., 2012; Rodrigues *et al*., 2019).

Indeed, in the genus *Pinus*, similar phenotypes or tolerances may be conserved by the effect of similar selective pressures; in this case we could expect strong phylogenetic signal (Contreras *et al*., 2017). Although studies related to climatic variables have increased recently, in the case of pines this information is disparate or lacks a phylogenetic framework. This lack of knowledge is even more noticeable in characteristics that are highly significant in determining growth, survival and ecological success of pine species such as DNA content, chemical soil properties, bark thickness and characteristics of needles and ovulate cones.

In some species of pines, there may be a reduction in DNA content, or in the dimensions of cones and needles as a response to drought or nutrient scarcity, or thicker bark could help mainly to protect against fire, but these relationships could be influenced by environmental and evolutionary factors (Joyner *et al*., 2001; Hernández *et al*., 2020; Shearman and Varner, 2021). A phylogenetic framework is needed to understand the complexity of these relationships, which could help us understand constraints or flexibility in adapting to changing environments.

In North American hard pines, there are large ecological differences along their distribution. The duration of the growing season shortens progressively with increasing latitude and elevation. From North America to Central America the genus *Pinus* thrives in warmer and drier environments. In California and Mexico, pines have experienced great geological and climatic changes in relatively recent geological time such that their evolutionary history has been linked to periods of intense volcanic activity (Farjon, 1996). Additionally, because of varied topography and climate in Mexico, natural populations of pines thrive in a range from wet, lowland tropical rainforest and montane tropical cloud forest to hot scrublands and snow-capped mountains, with heterogeneous edaphic conditions (Rzedowski, 2006).

A key assumption in comparative patterns is that variation is a product of population-level processes (Olson and Arroyo-Santos, 2015), and hence phylogenetic signal might be a result of different processes (Revell *et al*., 2008; Münkemüller *et al*., 2012). In this context the niche similarity test between pairs of species could help us reinforce the hypothesis of phylogenetic niche conservatism, determining whether niches are similar to those expected at random. Interestingly, sometimes the niche similarity test does not reflect phylogenetic relationships, and in these cases, we could expect that the current distribution of species is determined by an environmental component (Giraldo-Kalil *et al*., 2023), which could help us understand the ability to respond to a difference in selective pressures.

**Fig. 1.**
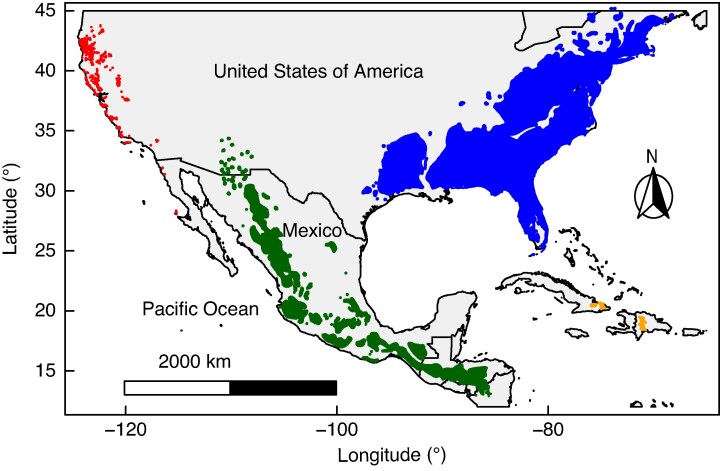
Geographical distribution (from Little, 1971) of the genus *Pinus* (Pinaceae) for the species included in this study. Blue indicates species from the eastern United Sates, red from the western United States, orange from the Caribbean and green from Mexico and Central America.

To test these assumptions, we used a group of hard pines distributed in the USA, Mexico, Central America and the Caribbean, *Pinus* subsection *Australes* Loudon, and the closely related *Attenuatae* clade (Fig. 1). The geographical distribution of these pines is presumably the result of migrations from North America toward lower latitudes during Oligocene cooling (Rzedowski, 2006; Donoghue, 2009). Our study was performed in three stages using a phylogenetic framework. First, we determined possible correlations between morphological and anatomical characteristics and climate to detect possible climatic gradients. Second, we assessed the phylogenetic signal in relevant climate and morphological and anatomical characteristics, tested different evolutionary models and performed an ancestral states reconstruction. Third, to corroborate the results of the comparative method, we performed niche similarity tests, and inferred differences in potential distribution through ecological niche modelling for each species. Our study provides evidence of strong phylogenetic signal in morphological and anatomical characters and chemical soil properties, and for phylogenetic niche conservatism.

## MATERIALS AND METHODS

We included 30 species from *Pinus* subsection *Australes* and the Attenuatae clade. The three species comprising the Attenuatae clade were treated as a separate subsection in the past (Price *et al*., 1998) but were merged into subsection *Australes* based on a well-supported sister relationship recovered in phylogenetic analyses of plastid DNA (Gernandt *et al*., 2005). However, a recent transcriptome-based study (Jin *et al*., 2021) recovered all members of subsection *Australes* except the Attenuatae as monophyletic and sister to subsection *Contortae*, with the Attenuatae sister to this larger group. The transcriptome results highlight uncertainty in the interrelationships among the Australes, Attenuatae and Contortae clades that requires further study. In most cases two or three samples per species were included, resulting in 72 individuals. Of these species we included three with their varieties: *P. radiata* var. *radiata* and *P. radiata* var. *binata*, *P. patula* var. *patula*, and *P. patula* var. *longipedunculata* and *P. greggii* var. *greggii*, and *P. greggii* var. *australis* (Supplementary Data Table S1). We included one sample from subsection *Sabinianae* (*Pinus coulteri*) as an outgroup (Willyard *et al*., 2021). The samples cover the geographical range of subsections *Australes* and *Attenuatae*, and they are distributed geographically in North America, Mexico, Central America and the Caribbean (Fig. 1). DNA was extracted from leaf tissue with the CTAB method (Doyle and Doyle, 1987) or from megagametophyte tissue with a Wizard Genomic Purification Kit (Promega, Madison, WI, USA). We measured DNA concentration with a Qubit fluorometer and Qubit dsDNA HS assay kit (Life Technologies, Carlsbad, CA, USA). To prepare libraries we used 100–500 ng of DNA for each sample.

### Assembly, alignment and phylogenetic relationships

The origin of genes used in target enrichment was an exonic study of *Pinus taeda* and *P. elliottii* (Neves *et al*., 2013). The preparation of libraries, target enrichment and sequencing preparation were done by Arbor Biosciences (Ann Arbor, MI, USA). Sequences were determined at different dates using an Illumina Hi-Seq 2500, 4000 or NovaSeq S4 with 100-, 125- or 150-bp paired ends (see Supplementary Data Table S1 for details). Demultiplexing was performed using barcode sequences and combined into paired fastq files (R1 and R2) for each sample. Reads were then trimmed using Trimmomatic v.0.39 (Bolger *et al*., 2014), retaining sequences with a minimum length of 36 bp.

The trimmed reads for 72 samples were assembled into individual files with HybPiper v.1.3.1. (Johnson *et al*., 2016), using Biopython (Cock *et al*., 2009). For the assignment of sequence reads to loci we initially had a dataset of 1117 genes. The pipeline implemented in HybPiper performs read sorting with the BWA method (Li and Durbin, 2010). We provided the nuclear exonic genes from the probe design step as references. Using the reads previously identified with BWA, the pipeline performed a *de novo* assembly with *SPAdes* v.3.10.1 (Bankevich *et al*., 2012) for each gene individually. If target sequences contain multiple exons of the same gene, these were concatenated into a single coding sequence, as HybPiper can detect intron sequences and either remove them to make gene assemblies or include them in supercontig assemblies.

The gene files were aligned with MAFFT v.7.505 (Katoh and Standley, 2013). We used two criteria for selecting our dataset of genes for phylogenetic inference: first, based on a visual check of the alignments, and second, requiring that all individuals were represented in the selected alignments. This step resulted in a dataset of 630 genes. With these genes we obtained a concatenated matrix with a total length of 420 192 bp, with 31 924 parsimony-informative sites. With this matrix we reconstructed phylogenetic relationships using the maximum likelihood method in IQ-TREE2 (Minh *et al*., 2020) with the model TVM+F+I+R6 chosen with Modelfinder (Kalyaanamoorthy *et al*., 2017) and 1000 ultrafast bootstrap replications with the function UFBoot (Hoang *et al*., 2018).

With the phylogenetic relationships obtained with IQTREE2 we inferred a species tree including terminal and internal branch lengths in substitution-per-site units using ASTRAL-IV (Zhang *et al*., 2018; Zhang and Mirarab, 2022; Tabatabaee *et al*., 2023). To obtain the species tree we used the bootstrap tree file as input using the *-a* option. The species tree was converted to a dated ultrametric tree using penalized likelihood in TREEPL (Smith and O’Meara, 2012). We fine-tuned divergence time priors using secondary calibration from a previous study of pines (Jin *et al*., 2021). This included a maximum age of ∼25 Ma and a minimum age of ∼20 Ma for the common ancestor to all clades, a maximum age of ∼20 Ma and a minimum age of ∼10 Ma for the *Attenuatae* clade (*P. attenuata*, *P. muricata*, *P. radiata* var. *radiata* and *P. radiata* var. *binata*), a maximum age of ∼20 Ma and a minimum age of ∼10 Ma for the clade with *P. taeda*, *P. serotina*, *P. glabra*, *P. rigida* and *P. pungens*, a maximum age of ∼15 Ma and a minimum age of ∼5 Ma for the clade of *P. elliottii*, *P. palustris*, *P. occidentalis* and *P. cubensis*, and a maximum age of ∼15 Ma and a minimum age of ∼5 Ma for pines from Mexico and Central America (*P. patula* var. *patula*, *P. patula* var. *longipedunculata*, *P. greggii* var. *greggii*, *P. greggii* var. *australis*, *P. tecunumanii*, *P. teocote*, *P. herrerae*, *P. lumholtzii*, *P. leiophylla*, *P. chihuahuana*, *P. pringlei*, *P. lawsonii*, *P. jaliscana*, *P. praetermissa*, *P. georginae*, *P. luzmariae*, *P. oocarpa* and *P. vallartensis*). The species tree and ultrametric tree are available at: https://github.com/jorgecruzn/JCN_tree_hardpines.

### Relationships between morphology and climate

Climate and soil properties might directly influence bark thickness and needle and ovulate cone dimensions as trees adapt to their surroundings. To infer possible responses to selective pressures within a phylogenetic framework, we tested the relationship between DNA content, bark thickness and needle and cone characters with climatic variables. We developed general linear models with a Poisson distribution in the cases where the response variable was the result of counts, i.e. number of needles per fascicle and number of needle resin canals (Bolker *et al*., 2009), using the *glm* function in R v.4.3.0. (R Development Core Team, 2023). For needle and cone length, bark thickness and DNA content we developed linear models with climatic variables. Only in those cases where we found significant evidence for correlations, we conducted phylogenetic generalized least squares (PGLS) analyses using the *gls* function in the *nlme* package in R (Pinheiro and Bates, 2000; Pinheiro and Bates, 2023).

### Phylogenetic signal

To test the retention or change of character states considering phylogenetic relationships, we assessed the phylogenetic signal in 17 continuous characters using two metrics, Pagel’s λ (Pagel, 1999) and Blomberg’s *K* (Blomberg *et al*., 2003). Seven of the 17 characters were climatic variables: *annual mean temperature* (*bio 1*), *maximum temperature of warmest month* (*bio 5*), *minimum temperature of coldest month* (*bio 6*), *temperature annual range* (*bio 7*), *annual precipitation* (*bio 12*), *precipitation of wettest month* (*bio 13*) and *precipitation of driest quarter* (*bio 17*). To obtain climate data for each species we compiled data from longitude and latitude of our collections and reviewed specimens in the Herbario Nacional de México (MEXU) and other available data for *P. muricata* (Téllez-Valdés *et al*., 2019). We complemented this information with presence records from the GBIF portal (https://www.gbif.org/; GBIF.org, 2024*a*, *b*, *c*, *d*, *e*, *f*, *g*, *h*, *i*, *j*, *k*, *l*). To clean these records, we eliminated those with taxonomic uncertainty, duplicates or outside the area of known geographical distribution. To avoid spatially autocorrelated overfitting, we eliminated points less than 5 km apart from one another with the package *spThin* for R (Aiello-Lammens *et al*., 2015). We retrieved climate information in raster format from Fick and Hijmans (2017) at a resolution of 2.5-minutes (∼5 km) using the *extract* function in the *raster* package in R (Hijmans, 2019). We assessed the phylogenetic signal in five morphological or anatomical characters that reflect the pines’ adaptations to optimize water conservation, defence mechanisms and reproductive success based on local climate. These were *number of needles per fascicle*, *needle length*, *number of resin canals*, *cone length* and *bark thickness*. Number of needles per fascicle, needle length and cone length were observed or measured directly from herbarium specimens available at MEXU (Supplementary Data Table S1). Bark thickness and number of resin canals were obtained from available literature (Martínez, 1992; Eckenwalder, 2009; Hernández *et al*., 2020). To assess the phylogenetic signal in four chemical soil properties [nitrogen (cg kg^–1^), pH, organic carbon (dg kg^–1^) and cation exchange capacity (mmol(c) kg^–1^)], we retrieved information available in raster format from the portal SoilGrids at a spatial resolution of 250 m (v.2.0, https://soilgrids.org/, accessed 22 December 2024; Poggio *et al*., 2021). Climate, chemical soil properties, needle length and cone length averaged by species were used for each species. To assess the phylogenetic signal in genome size (pg), we retrieved DNA C-values from the portal https://cvalues.science.kew.org/search/gymnosperm (accessed 17 December 2024). C-values represent the amount of DNA in a haploid nucleus (such as in a gamete) and are directly related to genome size. When the information was not available for reference, we used the standard conversion of 1 pg = ∼978 Mb (Doležel *et al*., 2003). Specifically, the mean weight of one nucleotide pair is 1.023 × 10^−9^ pg, and 1 pg of DNA would represent 1 pg = ∼978 Mb, and genome size (bp) = (0.978 × 10^9^) × DNA content (pg).

To complement our inferences on phylogenetic signal we tested three evolutionary models (Münkemüller *et al*., 2015). These models were chosen for species with temperate affinities that have diversified under shifting environmental conditions (Klečková *et al.,* 2023). The first, Brownian Motion, indicates a random walk where the differences among species are proportional to time as a result of a single evolutionary rate. In this model a continuous character evolves according to a suite of random steps in the absence of selection; it is typically used to represent neutral drift over macro-evolutionary timescales (Felsenstein, 1985; Münkemüller *et al.,* 2015). The second, Ornstein–Uhlenbeck (Hansen, 1997; Butler and King, 2004), describes a single evolutionary rate indicating few changes along the phylogeny, and suggesting stabilizing selection. This model has been proposed to model evolution toward a selective optimum in comparative analyses because it constrains the evolution in bounded phenotypic space (Boucher *et al*., 2014; Münkemüller *et al*., 2015). The third, the Early Burst model (Harmon *et al*., 2010), allows earlier changes, as expected for an adaptive radiation. Under this scenario, it is expected that climatic niches among sister species evolve rapidly with the changes close to the root of the phylogeny (Klečková *et al*., 2023). These models were compared using Akaike’s information criterion (AIC) and weighted AIC (AICw) to determine the best-fitting model. Those models with an AIC and ΔAIC greater than 2 were considered different (Burnham and Anderson, 2002). A good fit to the Ornstein–Uhlenbeck model was interpreted as evidence of phylogenetic niche conservatism, because it represents a constraint around an optimal value and is not just a function of time (Rodrigues *et al*., 2019).

To evaluate the influence of selection and genetic drift we compared variants of the Ornstein–Uhlenbeck model for two different groups: (i) North American and (ii) Mexican and Central American pines. We inferred the following parameters: optimum trait value (θ), selection strength (α) and the variance of the random component of trait evolution (σ^2^), representing genetic drift. We compared four variants of the Ornstein–Uhlenbeck models (Butler and King, 2004; Beaulieu *et al*., 2012). First, a general OU1 assuming a single adaptive optimum (θ) for a continuous character trait across the phylogeny with a single rate of adaptation or strength of selection (∝); second, an OU_M_ model that assumes a different optimum for each group but a single rate of stochastic motion (σ^2^; genetic drift) and strength of selection (∝); third, an OU_MV_ model allowing different optima for each group and variation in the rate of stochastic motion but with selection strength (∝) constant; and fourth, an OU_MA_ model allowing for variation in the strength of selection (∝) and a different optimum value but with stochastic motion rate constant (σ^2^). These models were fitted using the function OUwie in the R package OUwie (Beaulieu *et al*., 2012) and compared using AIC.

### Ancestral state reconstruction

To test whether pines rapidly changed their environmental preferences or morphology after divergence, we reconstructed ancestral states at nodes of the phylogeny from observed log values of climatic variables (except minimum temperature of coldest month), DNA content, morphoanatomical traits and chemical soil properties of individual species using the ultrametric tree with the function *contMap* in *phytools* (Revell, 2012).

### Niche overlap

To corroborate our hypothesis of phylogenetic niche conservatism and verify if there is a correspondence between the niche similarity and phylogenetic relationships, i.e. closely related species shared more niche similarity, we performed niche overlap tests among closely related and distantly related species. More similarity among closely related species would indicate that the variation observed is the product of past evolutionary processes and, conversely, more similarity among more divergent species would indicate that the variation is the product of current environmental conditions. We performed niche similarity tests for different pine species pairs distributed in North America, Mexico, Central America and the Caribbean. We estimated the niche overlap with Shoener’s *D* metric (Schoener, 1970) among 80 species pairs. Higher values of niche overlap (i.e. higher values of Shoener’s *D*) indicate more niche similarity in climatic tolerances. For this purpose, we generated four buffers surrounding the presence points previously obtained for pines distributed in (i) the western United States, (ii) (northeastern and) eastern United States, (iii) southeastern United States and the Caribbean, and (iv) Mexico and Central America. We used each buffer as a mask for 15 bioclimatic variables (i.e. *bio 1*, *2*, *3*, *4*, *5*, *6*, *7*, *10*, *11*, *12*, *13*, *14*, *15*, *16* and *17*). These bioclimatic variables were retrieved in raster format from Fick and Hijmans (2017) at a resolution of 2.5 min (∼5 km). We used the framework proposed by Warren *et al*. (2008) to test niche similarity within a multivariate environmental grid constructed from the first two axes of a principal components analysis (PCA) that summarized the 15 bioclimatic variables (Broennimann *et al*., 2012). We subsequently estimated Shoener’s *D*, and tested niche similarity for different species pairs using the function *ecospat* in R (Di Cola *et al*., 2017) with a special emphasis on comparisons between hard pines from Mexico and Central America versus hard pines from the United States and the Caribbean. The test generates a null distribution based on Shoener’s *D* values between a species and background points of another species. To determine the significance of the test, the null distribution is compared to the observed value.

### Ecological niche modelling

To visualize the climatic niche in geographical space for these pines and to evaluate possible differences in the centroids for each bioclimatic variable, we estimated the Grinellian niche for each taxon on climatic dimensions (i.e. climatic niche), except endemic species with restricted distribution and fewer than seven presence points (i.e. *P. jaliscana*, *P. vallartensis*, *P. georginae*, *P. pringlei* and *P. lawsonii*). We used the same presence points, calibration area (*M*) and climatic variables used for niche overlap. We assumed that fundamental niches were convex in shape, and they could be viewed as multidimensional ellipsoids. We estimated niche models using ellipsoids as implemented in Niche ToolBox 0.5.1.4 (Van Aelst and Rousseeuw, 2009; Osorio-Olvera *et al*., 2018), and then projected them on the geographical space. To evaluate the model, we used the partial receiver operating characteristic (partial ROC), which is a non-parametric indicator of model performance (Peterson *et al*., 2008, 2011). To avoid overfitting with bioclimatic variables, we omitted correlated variables (>0.8). With the retained variables for each species, we tested different combinations (3, 5 and 6) with the function *ellipsoid_selection* and selected the best model using the results of partial ROC. Finally, to infer possible divergences among species, we performed the Kruskal–Wallis tests among the values of centroids for five bioclimatic variables of each species (*bio 1*, *bio 2*, *bio 12*, *bio 14* and *bio 15*). A significant difference would indicate divergence among species; conversely, the absence of differences suggests niche conservatism among species.

## RESULTS

### Relationships between morphology and climate

The number of resin canals had a positive relationship with precipitation of wettest month, whereas needle length had a positive relationship with annual mean temperature and precipitation of wettest month. The relationship between DNA content and annual mean temperature was negative. These significant relationships (*P* < 0.05) also persisted after PGLS, suggesting a strong relationship of needle length and number of resin canals with climate. Although initially the analysis suggested a relationship between needles per fascicle and cone length with precipitation, and between DNA content and precipitation, these relationships were not significant after considering the phylogenetic effect (Supplementary Data Fig. S1, Table S3).

### Phylogenetic signals

Phylogenetic signal was strong for most characters (λ > 0.57, *P* < 0.05), but in two characters (soil organic carbon and cation exchange capacity) we found a weaker phylogenetic signal (λ < 0.36, *P* > 0.05). The phylogenetic signal measured with Blomberg’s *K* was strong (*K* > 0.68, *P* < 0.05) except for needle length, annual precipitation, precipitation of wettest month, nitrogen, organic carbon and cation exchange capacity in soil (*K* = 0.19–0.49, *P* > 0.05; Table 1). Ornstein–Uhlenbeck was the best model for seven characters (needles per fascicle, needle length, annual precipitation, precipitation of wettest month, nitrogen, organic carbon and cation exchange capacity, ΔAIC > 2). Early Burst was the best model for DNA content, temperature annual range and precipitation of driest quarter (ΔAIC > 2).

**Table 1.** Values of Pagel’s λ and Blomberg’s *K* in hard pines inferred for soil, climate and morphological and anatomical variation. Values in bold are statistically significant.

Character	Pagel’s λ	*P*-value	Blomberg’s *K*	*P*-value
Needles per fascicle	0.77498	**0**.**00048**	0.77476	**0**.**01900**
Needle length	0.99993	**0**.**00000**	0.37007	0.61800
Number of resin canals	0.99993	**0**.**00119**	0.88532	**0**.**00200**
Cone length	0.90155	**0**.**00378**	1.42061	**0**.**00100**
Bark thickness	0.97320	**0**.**00003**	2.46485	**0**.**00100**
DNA content	0.99993	**0**.**00000**	2.87176	**0**.**00100**
Annual mean temperature	0.91276	**0**.**01294**	0.89790	**0**.**00600**
Maximum temperature of warmest month	0.85406	**0**.**03160**	0.68341	**0**.**01700**
Minimum temperature of coldest month	0.95582	**0**.**00056**	0.89393	**0**.**00600**
Temperature annual range	0.99993	**0**.**00000**	4.10164	**0**.**00100**
Annual precipitation	0.62403	**0**.**00839**	0.45778	0.49600
Precipitation of wettest month	0.57644	**0**.**00365**	0.49724	0.26400
Precipitation of driest quarter	0.98173	**0**.**00000**	1.81084	**0**.**00100**
Cation exchange capacity	0.00006	1.00000	0.36045	0.60700
Nitrogen in soil	0.72595	**0**.**00499**	0.46261	0.37400
Soil organic carbon	0.360113	0.172465	0.19754	0.94800
Soil pH	0.893884	**0**.**00031**	0.95332	**0**.**00100**

Brownian Motion was the best model for cone length and minimum temperature of coldest month (ΔAIC > 2). For five characters (number of resin canals, bark thickness, annual mean temperature, maximum temperature of warmest month and pH in soil), we could not discriminate between Brownian Motion and Ornstein–Uhlenbeck (ΔAIC < 2, Supplementary Data Table S4). Overall, these results indicate that random walk and stabilizing selection had a significant effect on the evolution of characters along the phylogeny.

When comparing different parameters of Ornstein–Uhlenbeck, the best model based on ΔAIC was OU_MA_ for needles per fascicle, precipitation of wettest month, nitrogen and organic carbon in soil, indicating higher complexity with differences in the adaptive optima and evolutionary rates. The model with a different optimum, OU_M_, was the best model for annual precipitation and cation exchange capacity in soil, but for needle length, OU_MV_ was the best model, indicating differences in the strength of stabilizing selection (Supplementary Data Table S5).

### Ancestral state reconstruction

**Fig. 2.**
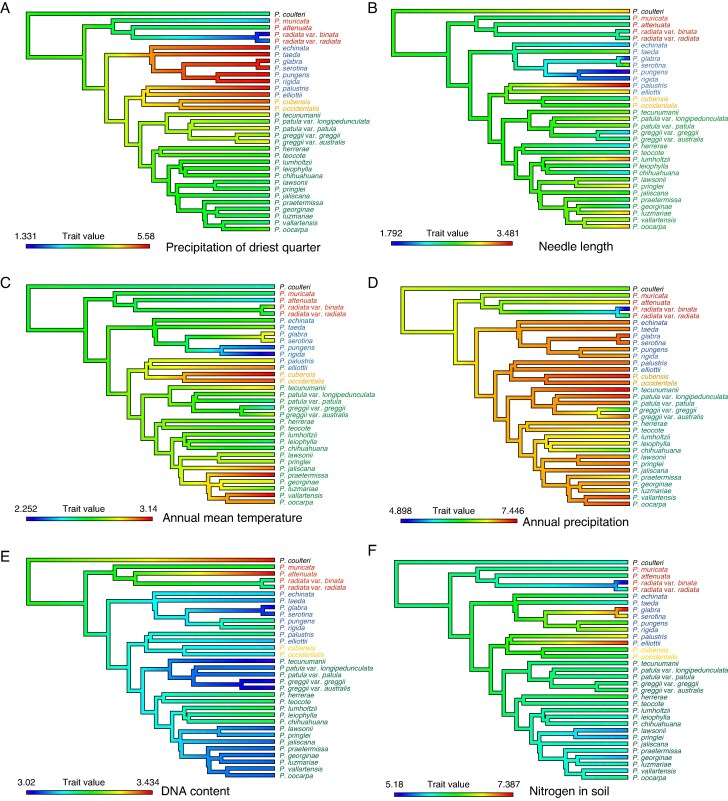
Representative ancestral state reconstruction for six characters: (A) precipitation of driest quarter; (B) needle length; (C) annual mean temperature; (D) annual precipitation; (E) DNA content; (F) nitrogen in soil (see also Supplementary Data Fig. S2). The colors of the terminal taxa are as follows: blue for the eastern United Sates, red for the western United States, orange for the Caribbean taxa, and green for the pines from Mexico and Central America.

The number of needles per fascicle, and number of resin canals per needle converged toward lower values in pines from the eastern United States, and higher values in Mexican pines (*P. luzmariae*, *P. georginae*, *P. oocarpa* and *P. vallartensis*). For the variables precipitation of wettest month, nitrogen, organic carbon, pH in soil, needle length and cone length we found few changes along the phylogeny. *Pinus palustris* and *P. elliottii* shared the highest values of needle length and cone length, and *P. radiata* var. *binata* had autapomorphies in organic carbon, nitrogen and cation exchange capacity in soil. Annual mean temperature, maximum temperature of warmest month, annual precipitation, pH in soil and number of resin canals had changes similar to expected under Brownian Motion along the phylogeny. We found interesting synapomorphies for minimum temperature of coldest month in *P. pungens* and *P. rigida*, which had the lowest values, and *P. occidentalis* and *P. cubensis*, which had the highest values. For DNA content, temperature annual range and precipitation of driest quarter we found few changes along the phylogeny, with an early divergence. For precipitation of driest quarter, pines from the eastern United States and the Caribbean (*P. echinata*, *P. serotina*, *P. glabra*, *P. taeda*, *P. elliottii*, *P. palustris*, *P. occidentalis* and *P. cubensis*) converged on higher values (Fig. 2; Supplementary Data Fig. S2).

### Niche overlap

**Fig. 3.**
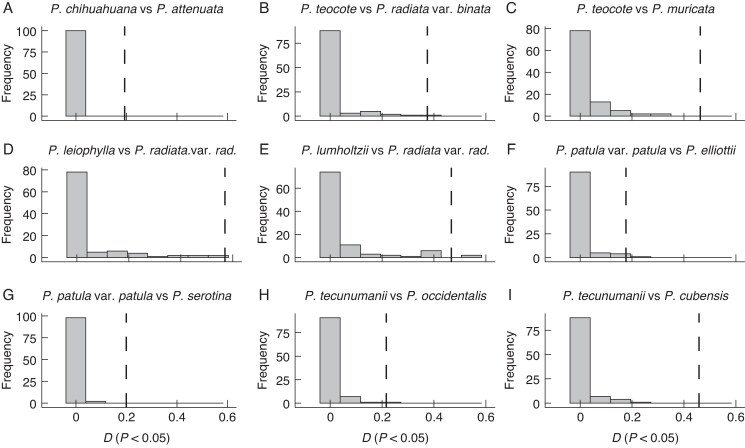
Histograms depicting the background similarity test for niche overlap of representative species pairs of hard pines using Shoener’s *D*. Null distributions of background niches available for each species are shown as grey bars; observed overlap values are represented by black dashed lines. In these cases, the overlap is higher than expected (*P* < 0.05).

In the 80 comparisons performed we found niche similarity in all cases, i.e. the niches were not different. However, the values of Shoener’s *D* ranged from 0 to 0.56 with a mean of 0.114 (s.e. = 0.156). In 31 comparisons (38.75 %) the values of niche overlap ranged from 0 to 0.017; these values were as expected (*P* > 0.05). Here we made comparisons between pines from lower latitudes (especially Mexican pines) and pines from higher latitudes (*P. rigida*, *P. taeda*, *P. pungens*, *P. glabra*, *P. echinata*, *P. elliottii* and *P. palustris*). In 23 comparisons (28.75 %) we found more similarity than expected by chance (*P* < 0.05). Surprisingly, in these comparisons Mexican pines at lower latitudes shared similarity with pines from North America or the Caribbean. Three closely related species (*P. patula* and *P. greggii*, each with their respective varieties, and *P. tecunumanii*) shared more similarity than expected with the more phylogenetically divergent species *P. serotina*, *P. elliottii*, *P. cubensis* and *P. occidentalis*. Another group of closely related species located in Mexico (*P. chihuahuana*, *P. leiophylla*, *P. teocote*, *P. herrerae* and *P. lumholtzii*) shared more similarity with phylogenetically divergent species from the western United States (*P. attenuata*, *P. radiata* with its two varieties and *P. muricata*). Other comparisons with more similarity than expected were between divergent species *P. oocarpa* versus *P. cubensis*, and *P. luzmariae* versus *P. muricata* (Fig. 3; Supplementary Data Fig. S3 and Table S6).

### Ecological niche modelling

**Fig. 4.**
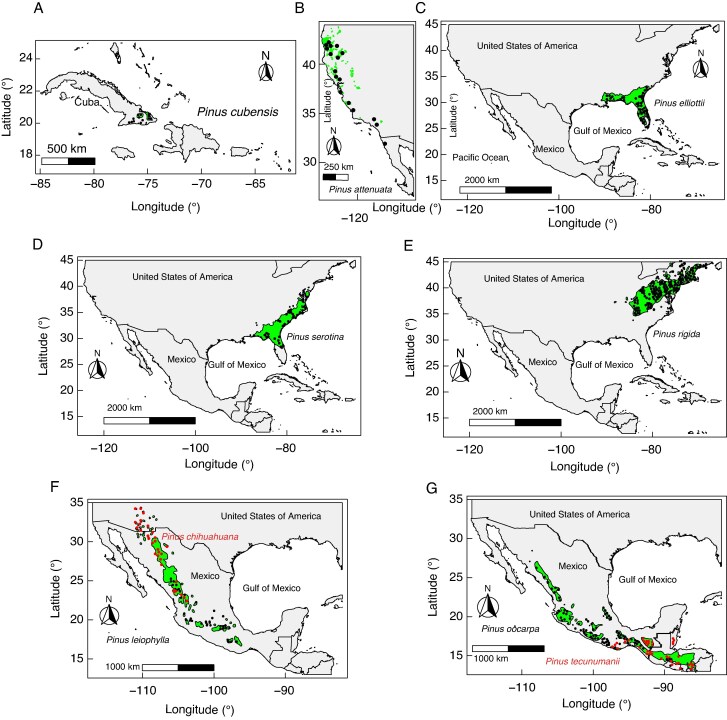
Geographical distribution (green; from Little, 1971) of the genus *Pinus* (Pinaceae) in the United States, Mexico, the Caribbean and Central America. The points represent locations included for ecological niche modelling in this study. This figure shows the geographical distribution of a representative subset of the studied species, selected for their relevance to the results.

All models for each species were better than null random expectations according to partial ROC tests (Supplementary Data Table S7). The five main bioclimatic variables to describe the climatic niche were annual mean temperature (*bio 1*), mean diurnal range (*bio 2*), annual precipitation (*bio 12*), precipitation of driest month (*bio 14*) and precipitation seasonality (*bio 15*). We did not find significant differences in centroids for these bioclimatic variables according to the Kruskal–Wallis test (*P* > 0.05). Specific bioclimatic variables were important for climatic niche in some species: minimum temperature of coldest month (*bio 6*) in *P. radiata* var. *binata*, precipitation of wettest quarter (*bio 16*) and mean temperature of coldest quarter (*bio 11*) in *P. attenuata*, *P. greggii* var. *australis* and *P. radiata* var. *radiata*, and precipitation of driest quarter (*bio 17*) in *P. herrerae*. When we projected our models onto geographical space, some species showed a restricted geographical distribution and shared specific habitats, for instance *P. muricata* and *P. radiata* var. *radiata* along the West Coast of the United States, *P. occidentalis* and *P. cubensis* in the Caribbean islands, and *P. greggii* var. *australis* in the mountains of eastern Mexico. Conversely, species such as *P. palustris*, *P. oocarpa*, *P. chihuahuana* and *P. tecunumanii* had a wider potential distribution, indicating large differences in the geographical space available for each species (Fig. 4; Fig. S4).

## DISCUSSION

**Fig. 5.**
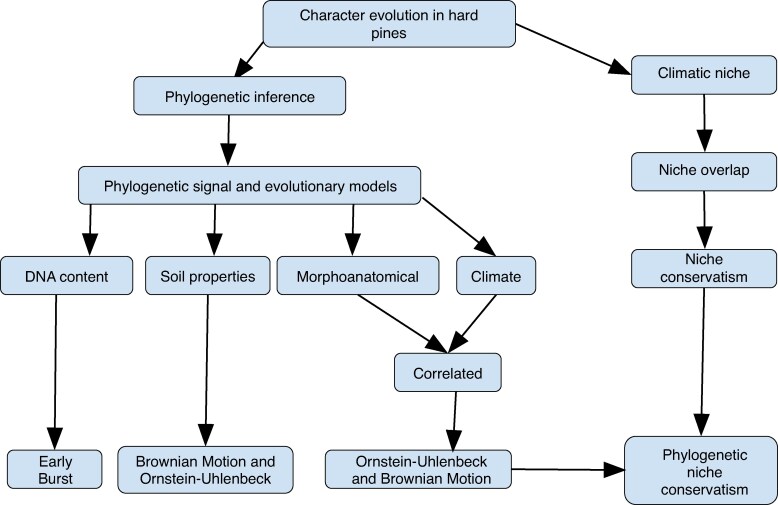
Schematic diagram summarizing the main findings in this study for hard pines in the United States, Mexico, the Caribbean and Central America.

Understanding the processes associated with the retention of characters is key for understanding the diversification of hard pines in lower latitudes under tropical conditions. We found a clinal relationship between needle length and temperature and precipitation, and between DNA content and temperature (Supplementary Data Fig. S1), indicating that historically the environmental component was relevant in the evolutionary history of these species (Davis and Shaw, 2001; Burleigh *et al*., 2012). The phylogenetic signals best fit two models, Ornstein–Uhlenbeck and Brownian Motion, indicating the action of stabilizing selection and random walk in the divergence of these pines (Fig. 5). This result, together with the presence of clines, suggests the possibility of different optima across the geographical distribution of these species (Kujala *et al*., 2023). DNA content, precipitation of driest quarter and temperature annual range best fit an Early Burst model, indicating that they resemble a rapid initial evolution that decelerated over time. We found niche similarity among these pine species, but in some cases, there was more than expected. This similarity was not related to phylogenetic relationships, indicating that the pattern of similarity is related to the current environmental variation across their geographical ranges. We did not find differences in the centroids of bioclimatic variables, which means that the niches are not different between species, supporting phylogenetic niche conservatism, but we found large differences in the available geographical space (Fig. 4; Fig. S4), indicating constraints in the geographical distribution of some species, again suggesting the importance of environmental variation in their distribution. Overall, our results support conservatism, with a response to the environment throughout the evolution of these pines (Fig. 5).

### Relationships between climate and morphology and phylogenetic signal

The strong phylogenetic signal in morphological characters λ (∼1) and the fluctuation in values of *K* (≥1) indicate that random walk is not the only possible explanation for the evolution of these characters. The correlations indicated that in wetter and hotter climates hard pines have more resin canals, longer needles and thicker bark, indicating that these characters have an adaptive response. The responses to growth associated with temperature and precipitation in conifer species has been observed in radial growth, in phenological traits (Nishimura and Laroque, 2011; Kujala *et al*., 2023) and in functional traits in oaks from the southeastern United States that co-occur, sharing similar photosynthetic rates, water-use efficiency and nitrogen content in similar environments (Cavender-Bares *et al*., 2018). The interaction of climate with morphological characters is frequent in pines (Cole *et al*., 2008), although the number of resin canals also could be a defence mechanism for insect pathogens (Cole *et al*., 2008; Huang *et al*., 2016). These responses are not entirely influenced by the environment. Interestingly, the relationships are strongly constrained by phylogeny (Supplementary Data Fig. S1; Table S3). Hence, although these responses could be detected over a short time period, at a macroevolutionary scale the divergence in one trait could covary with other traits and persist for many generations, even for millions of years (Schluter, 1996; Rombaut *et al*., 2022). If we consider that the diversification in this pine clade occurred during the Miocene (∼20 Ma; Jin *et al*., 2021), this relationship implies that these characters have not evolved independently. If these species have been under similar selective pressures for many generations (>100), then morphological convergence may be related to local adaptation (Kremer and Le Corre, 2012).

Needle characters are influenced by site characteristics due to a significant contribution of phenotypic plasticity; thus, in drier sites, needles tend to be shorter (López *et al.,* 2010). The positive relationship between needle size and precipitation has implications for the functional characters of needles. Longer needles involve an increased investment in mechanical tissue, which helps needles withstand temperature fluctuations, maintain structural rigidity to support snow loads, and protect xylem and phloem tissues for efficient water and nutrient transport. This is particularly important because longer needles require a greater water supply (Wang *et al*., 2019). Additionally, water availability is closely linked to the production of defensive compounds in resin ducts of conifers. Drought conditions could modulate the growth–defence trade-off by delaying tree growth while enhancing the production of resin-based defences (Saracino *et al.,* 2017; Vazquez-Gonzalez *et al.,* 2020).

A possible explanation for strong phylogenetic signal and no correlations with climate when considering the phylogenetic relationships (*K* > 1, Table 1;  Supplementary Data Table S3) for the number of needles per fascicle, bark thickness and cone length is that these characters are integrated with other ecological characters such as seed size in the case of cone length, or constraints of development in needles (Muir *et al*., 2023). A classic example of developmental integration is allometry, which may constrain phenotypic divergence at a macroevolutionary scale. Allometry in some cases may be aligned with the direction of selection, as in the case of reproductive organs, and size and density of stomata in leaves, even acting against maladaptive trait combinations; however, pleiotropy cannot be discarded as influencing the direction of phenotypic evolution (Pélabon *et al*., 2014; Muir *et al*., 2023).

Regarding the different optimal values between groups (North America and Mexico and the Caribbean pines) in needles per fascicle and needle length (Supplementary Data Table S5), the ancestral state reconstructions (Fig. 2;  Fig. S2) for morphological and anatomical characteristics indicate differences in historical adaptations throughout the evolutionary history of Mexican pines that have colonized tropical regions (*P. oocarpa*, *P. luzmariae*, *P. lawsonii*, *P. georginae*, *P. vallartensis*, *P. praetermissa* and *P. tecunumanii*) with more needles per fascicle and a greater number of resin canals.

### Phylogenetic signals in soil

Unlike morphological and anatomical characters, chemical soil properties had moderate to strong phylogenetic signal (Table 1). In three properties (cation exchange capacity, nitrogen and organic carbon), the best fit was the Ornstein–Uhlenbeck model (Supplementary Data Tables S4 and S5), indicating few changes along the phylogeny attributable to stabilizing selection (Fig. S2). In two characters, nitrogen and organic carbon, we had different optimal values (OU_MA_) between groups (North America, and Mexico and the Caribbean). The strength of stabilizing selection (α) could not be determined exactly, possibly because we had a small dataset (*n* = 33), which could be insufficient (Beaulieu *et al*., 2012). However, stabilizing selection was not the only explanation for maintaining the phylogenetic signal in chemical properties; for pH, random walk had an important contribution. Stabilizing selection indicates a trend toward an optimum with little variation. However, simultaneously, the contribution of random walk might be indicative of significant differences in species that have evolved in isolation. A similar phenomenon has also been suggested in other temperate-affinity taxa (Klečková *et al*., 2023).

### Phylogenetic signals and minor changes in environmental tolerances

We found strong phylogenetic signals in climatic variables. Signal in two variables related to precipitation (annual precipitation and precipitation of wettest month) indicated a considerable strength of selection and similarity of the optimum values between North American, and Mexican and Caribbean pines (OU_M_ in annual precipitation) and an important role of evolutionary history for both groups (North America, Mexico and the Caribbean) in precipitation of wettest month (OU_MA_). In contrast, the Brownian Motion model best explained three characters related to temperature, indicating that stabilizing selection was weak, and random walk better explains the variation across the phylogeny (Supplementary Data Table S5). These results support phylogenetic niche conservatism in this clade of hard pines. Simultaneously, the ancestral state reconstructions (Fig. 2;  Fig. S2) indicate that Mexican pine species such as *P. greggii*, *P. patula*, *P. chihuahuana*, *P. leiophylla* and *P. oocarpa* have thrived in warmer and drier conditions.

### Phylogenetic signals and Early Burst

Interestingly, in three characteristics, DNA content, precipitation of driest quarter and temperature annual range, Early Burst was the best model, reflecting an adaptive radiation scenario in which the characters change early, but over time the evolutionary rates slow and stabilizing selection becomes stronger. This finding of elevated rates of genome size in the diversification of pines has been mentioned previously (Burleigh *et al*., 2012). Although the relationship between genome size and latitude or ecological factors could be controversial (Hall, 2000; Joyner *et al*., 2001; Grotkopp *et al*., 2004), we found a negative correlation with annual mean temperature, suggesting the possibility of an adaptive process. Essentially, however, our results suggest that differences in genome size for hard pines are more related to evolutionary history, where despite their large genomes, pines still maintain slow rates of evolution compared to other plant lineages, which is indicative of their high degree of genome stability (Joyner *et al*., 2001).

### What insights do these three models provide?

There is an interesting interplay of these three evolutionary models (Ornstein–Uhlenbeck, Brownian Motion and Early Burst). Ornstein–Uhlenbeck points to an optimum in the traits analysed, indicating a low phenotypic disparity. In line with this, previous studies provide evidence of reticulation and cryptic diversity for these pines (Gernandt *et al*., 2018; Cruz-Nicolás *et al*., 2024*a*, *b*). The Brownian Motion model points to a contribution from random walk following divergence resulting in a moderate number of species. Although less frequently, an Early Burst model (i.e. adaptive radiation) also emerged as a possible explanation. Under these circumstances two fundamental axes, the low phenotypic disparity and the moderate number of species, support the expectation under a non-adaptive radiation in this clade of hard pines (Czekanski-Moir and Rundell, 2019; Martin and Richards, 2019). Their niche conservatism and geographical isolation also support this idea (Figs 3 and 4, Supplementary Data Figs S3 and S4).

### Niche overlap

We found a similarity of climatic niche among hard pines but large disparities in the values of niche overlap (Supplementary Data Table S6; Fig. 3), reinforcing the differences in selective pressures previously observed with morphology and climate. Niche similarity is expected under phylogenetic niche conservatism. We had low overlap (Shoener’s *D*), especially in those comparisons among hard pines from southern latitudes with more tropical conditions (*P. tecunumanii*, *P. oocarpa* and *P. leiophylla*), versus pines from northern latitudes with cooler climates (*P. echinata*, *P. glabra* and *P. rigida*). However, we found more similarity than expected between two phylogenetically divergent groups, one including *P. attenuata*, *P. muricata* and *P. radiata*, and the other including *P. oocarpa*, *P. luzmariae*, *P. chihuahuana*, *P. leiophylla*, *P. lumholtzii*, *P. herrerae* and *P. teocote*. These pines share similar needle length, bark thickness, number of resin canals and cation exchange capacity, indicating similar selective pressures with strong niche conservatism. These patterns of niche similarity did not reflect the phylogenetic relationships among species, and they could be attributed to the current environmental variation across the geographical ranges of the species, highlighting the importance of environmental variation (Giraldo-Kalil *et al*., 2023).

High ecological similarity in divergent groups is a possible outcome of convergent evolution. Convergence in ecological characteristics among North American hard pines appears to be an important factor in the evolutionary history of this group. In this case, convergence did not produce strict analogues because they have diverged but simultaneously share phylogenetic niche conservatism (Mccune, 1988). It is possible that niche conservatism has promoted allopatric speciation through population dispersal and isolation during past climatic fluctuations, as in other temperature groups, reinforcing the possibility of non-adaptive radiation for these pines (Klečková *et al.,* 2023; Martin and Richards, 2019).

In other cases we found higher overlap among divergent species with tropical affinities, such as *P. patula* var. *patula* with *P. serotina*, *P. elliottii* with *P. occidentalis*, *P. greggii* var. *australis* with *P. elliottii*, *P. tecunumanii* with *P. cubensis* and *P. occidentalis* and *P. oocarpa* with *P. cubensis* (Fig. 3;  Supplementary Data Table S6). Two notable features in some species are faster growth and the ability to survive in degraded soils. These traits have favoured the use of species such as *P. greggii* in forest plantations for commercial or protection purposes and breeding programmes (López-Upton *et al*., 2004). Previous studies have found changes in the evolutionary rate in some species of this group and correlations between single nucleotide polymorphisms and abiotic factors (Alfonso *et al*., 2020; Peláez *et al*., 2020; Cruz-Nicolás *et al*., 2024*a*, *b*). The presence of these characteristics in species such as *P. patula*, *P. greggii* and *P. tecunumanii* make them strong candidates for testing hypotheses of local adaptation in a context of global warming, or to perform genome annotation or make linkage maps. These kinds of studies would be useful as references for further population genetics and phylogenomic studies of Mexican and Central American pines.

### Ecological niche modelling

The absence of differences among the centroids for each bioclimatic variable (*P* > 0.05) also supported phylogenetic niche conservatism. However, with the results of the ecological niche modelling projected on geographical space we observed that those species with convergence in number of resin canals, nitrogen and cation exchange capacity in soil (*P. muricata*, *P. attenuata*, *P. radiata* var. *radiata*, *P. patula* and *P. greggii*) also had a more restricted distribution. The phenotypic variation in the number and position of resin canals might act as defences against herbivores or pathogens and are correlated with environmental gradients (Moreira *et al*., 2014). In our case we found that precipitation of wettest quarter and mean temperature of coldest quarter were important variables for inferring the climatic niche in these species; therefore, convergence in the position of resin canals might be related to defence mechanisms, but this should be tested experimentally (e.g. Moreira *et al*., 2014). In addition, the climatic niche does not consider relevant factors such as the presence of mycorrhizae in the soil for the case of pines and competition with other organisms limiting their geographical distribution.

### Diversification under phylogenetic niche conservatism

Overall, the results for phylogenetic signal, evolutionary models, reconstruction of ancestral states and niche similarity tests support the hypothesis of phylogenetic niche conservatism in these North American hard pines. Phylogenetic niche conservatism has allowed transitions into the tropics (e.g. *P. oocarpa*, *P. luzmariae*, *P. cubensis* and *P. occidentalis*). According to Jin *et al*. (2021), the diversification measured as number of species in this clade of hard pines occurred in the last ∼20 Myr. This is slightly more recent than in North American oaks but slightly older than Mexican firs (Cavender-Bares *et al*., 2018; Hipp *et al*., 2018; Cruz-Nicolás *et al*., 2024b). The common denominator in these cases is niche conservatism; however, diversification in this clade of pines has been greater at more southerly latitudes. Also, we found more similarity in species that are more divergent phylogenetically, indicating that the current environment plays a key role in their morphological variation. Simultaneously, the presence of clines suggests that historically the response to the environment has been a relevant component in the variation of these pines.

In conclusion, our study supports niche conservatism consistent with phylogenetic niche conservatism, although there was more similarity in some species that have evolved under similar selective pressures independent of phylogenetic relationships. Most evaluated characters (climatic, morphological, anatomical and soil chemical properties) showed a phylogenetic signal. However, no single evolutionary model can fully explain trait divergence. Depending on the specific trait, divergence might result either from constraint toward an adaptive optimum (stabilizing selection) or from random changes over time (random walk). We further observed an Early Burst diversification pattern for DNA content. The interaction of these evolutionary forces suggests a potential case of non-adaptive radiation in this clade of hard pines.

## Supplementary Material

mcaf147_Supplementary_Data

## SUPPLEMENTARY DATA

Supplementary data are available at *Annals of Botany*  online and consist of the following. Table S1. Collection information for material included in this study. Herbarium codes: MEXU = Herbario Nacional de México, OSC = Oregon State University. Table S2. Mean values for variables evaluated in this study. Table S3. Results of general linear models (needles per fascicle and number of resin canals) and linear models for DNA content, morphological and anatomical characters, with climate variables and PGLS. Table S4. AIC, delta AIC and AICw values for model fit of characteristics of hard pines. Table S5. Comparison among the different variations of OU models employed to test differences among North American, and Mexican and Caribbean pines. OU1: single optimum Ornstein–Uhlenbeck; OUm: Ornstein–Uhlenbeck with different species for each region; OUmv: Ornstein–Uhlenbeck assuming different state means in sigma square; OUma: Ornstein–Uhlenbeck assuming multiple alpha. Table S6. Results of overlap (*D*) among hard pine species. Table S7. Values of partial ROC, *P*-value, variables and centroids used for different ecological niche modelling using ellipsoids in hard pines. Fig. S1. Representative relationships among the number of resin canals, needle length, cone length and DNA content with different climate variables in hard pines. The grey shaded areas represent the 95 % confidence interval. Fig. S2. Ancestral state reconstruction for morphological characters, anatomical characters and climatic variables in hard pines obtained with *contMap.* All variables are shown in a logarithmic scale except for minimum temperature of coldest month. The colours of the terminal taxa are as follows: blue for the eastern United Sates, red for the western United States, orange for the Caribbean taxa, and green for the pines from Mexico and Central America. Fig. S3. Histograms depicting background similarity test for niche overlap of hard pine species pairs using Shoener’s *D*. Null distributions of background niches available for each species are shown as grey bars; observed overlap values are represented by black dashed lines. The significance value is indicated in parentheses. Fig. S4. Maps of potential distribution projected on the geography using ellipsoids for representative species of the genus *Pinus* in the United States, Mexico and Central America.
