# Supplementary material for: A combination of stabilizing selection and random walk are associated with phylogenetic signal in hard pines
Source: Ann Bot. 2025 Jul 16;136(4):821–36. doi: 10.1093/aob/mcaf147 (PMC12464948; doi:10.1093/aob/mcaf147)
Supplement: mcaf147_Supplementary_Data [file mcaf147_supplementary_data.zip › Suppl_Figures27jun25.docx]

**A combination of stabilizing selection and random walk are associated with phylogenetic signal in hard pines**

Jorge Cruz-Nicolás and David S. Gernandt

**Supplementary figures**


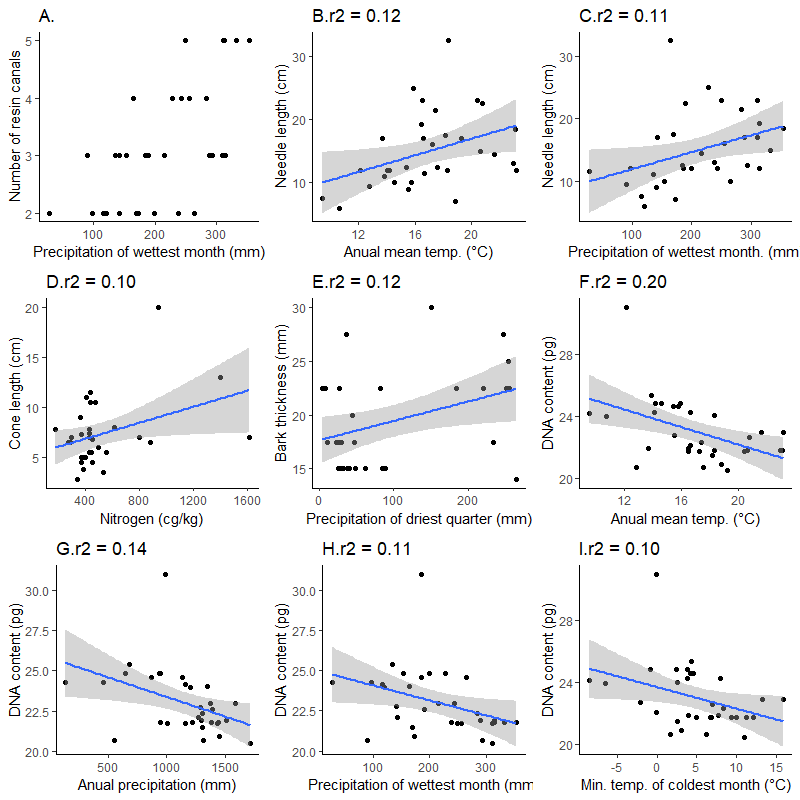


**Figure S1**. Representative relationships among number of resin canals, needle length, cone length and DNA content with different climate variables in hard pines. The gray shaded areas represent the 95% confidence interval.


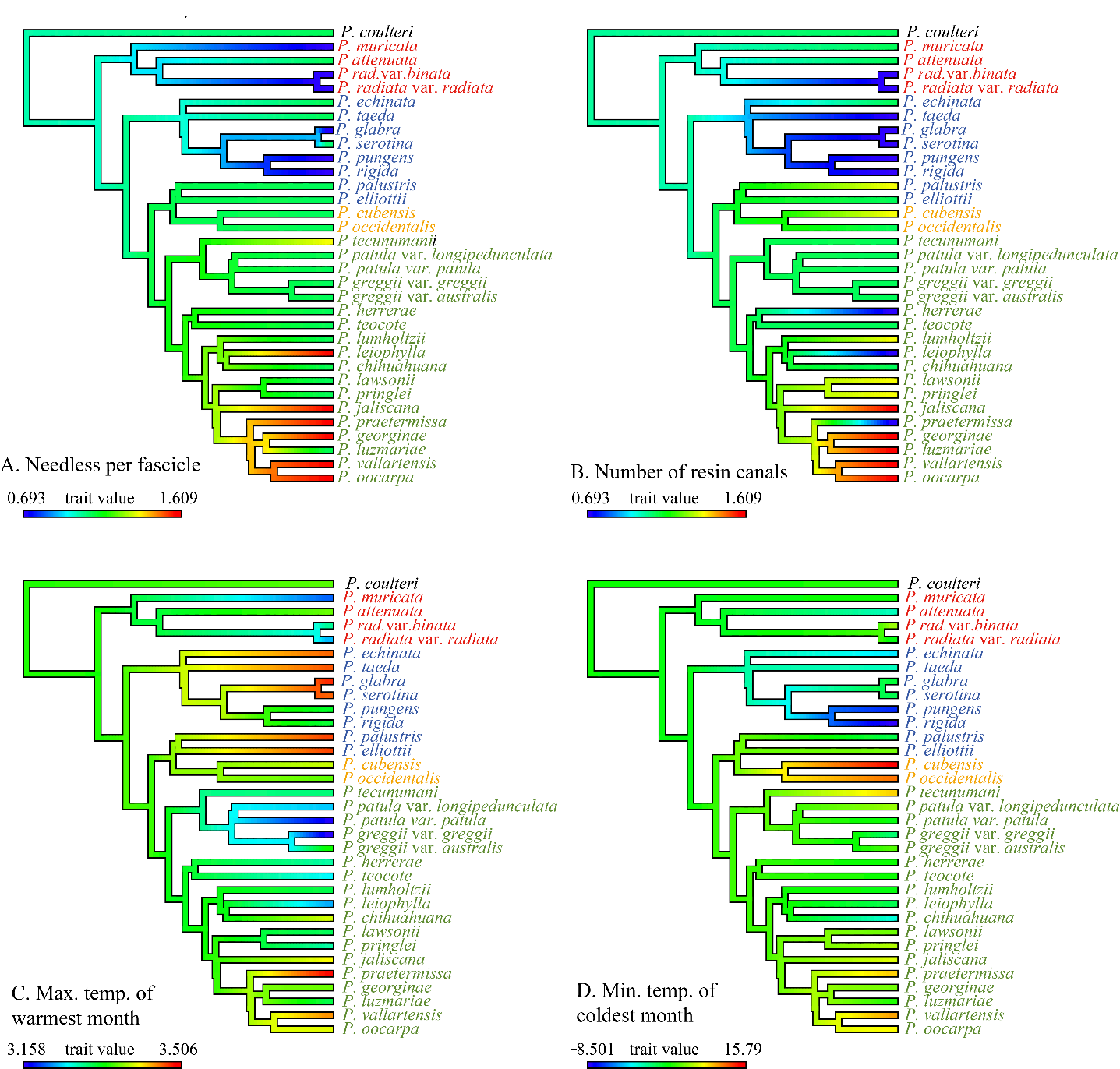


**Figure. S2**. Ancestral state reconstruction for morphological, anatomical characters and climatic variables in hard pines obtained with *contMap.* All variables are showed in logarithmic scale except minimum temperature of coldest month. The colours of the terminal taxa are as follows: blue for the eastern United Sates, red for the western United States, orange for the Caribbean taxa, and green for the pines from Mexico and Central America.


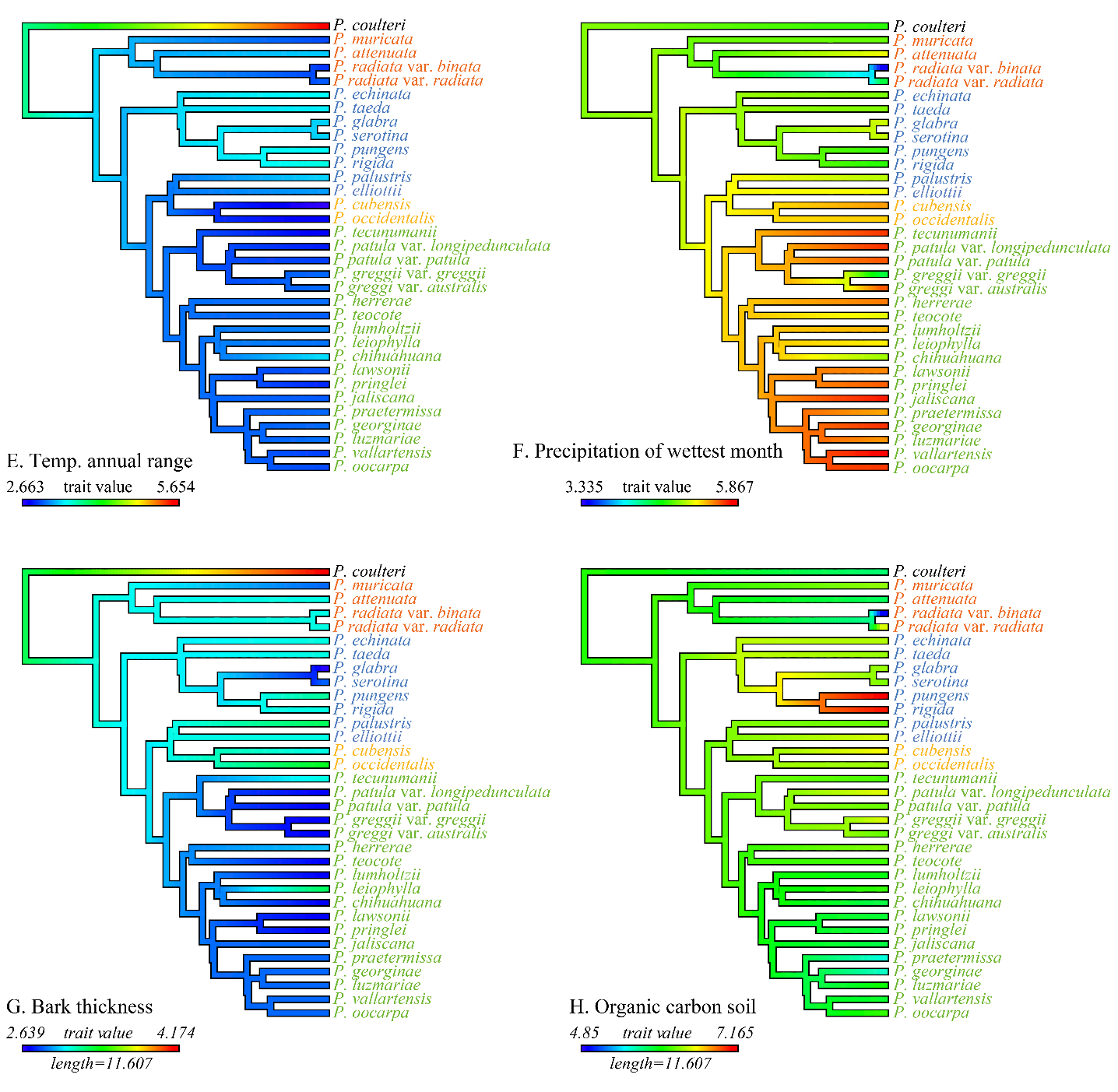


**Figure. S2**. Ancestral state reconstruction for morphological, anatomical characters and climatic variables in hard pines obtained with *contMap.* All variables are showed in logarithmic scale except minimum temperature of coldest month. The colours of the terminal taxa are as follows: blue for the eastern United Sates, red for the western United States, orange for the Caribbean taxa, and green for the pines from Mexico and Central America (continued…).


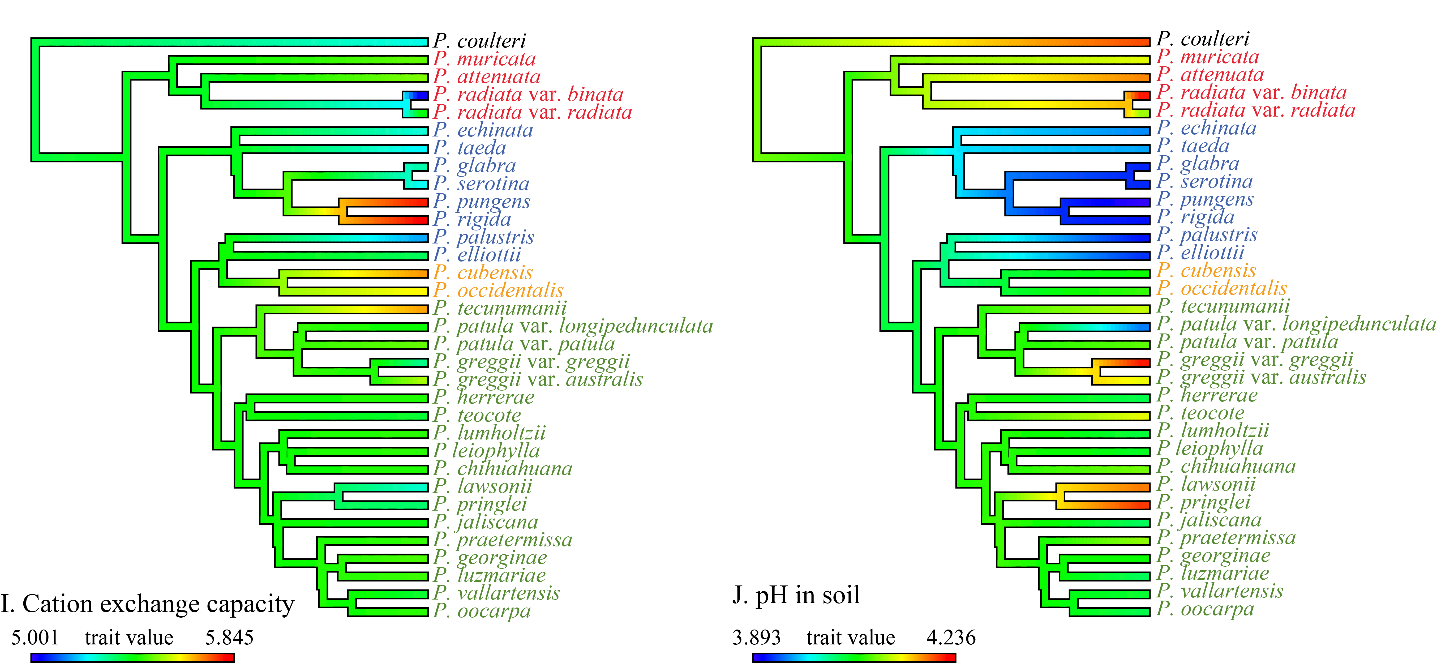


**Figure. S2**. Ancestral state reconstruction for morphoanatomical characters and climatic variables in hard pines obtained with *contMap.* All variables are showed in logarithmic scale except minimum temperature of coldest month. The colours of the terminal taxa are as follows: blue for the eastern United Sates, red for the western United States, orange for the Caribbean taxa, and green for the pines from Mexico and Central America (continued…).


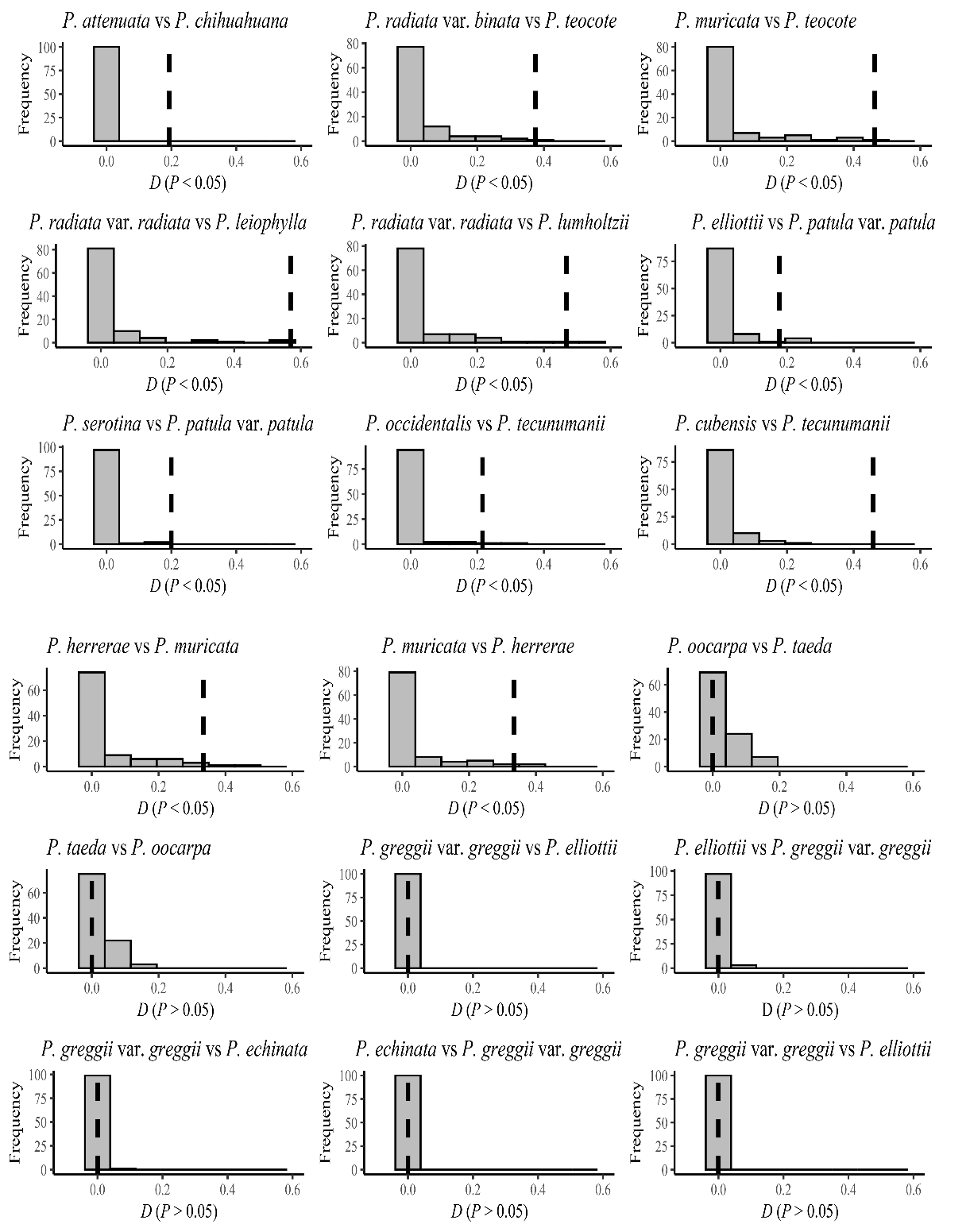


**Figure S3**. Histograms depicting background similarity test for niche overlap of hard pines species pairs using Shoener’s *D*. Null distributions of background niches available for each species are shown as gray bars; observed overlap values are represented by black dashed lines. The significance value is indicated in parentheses.


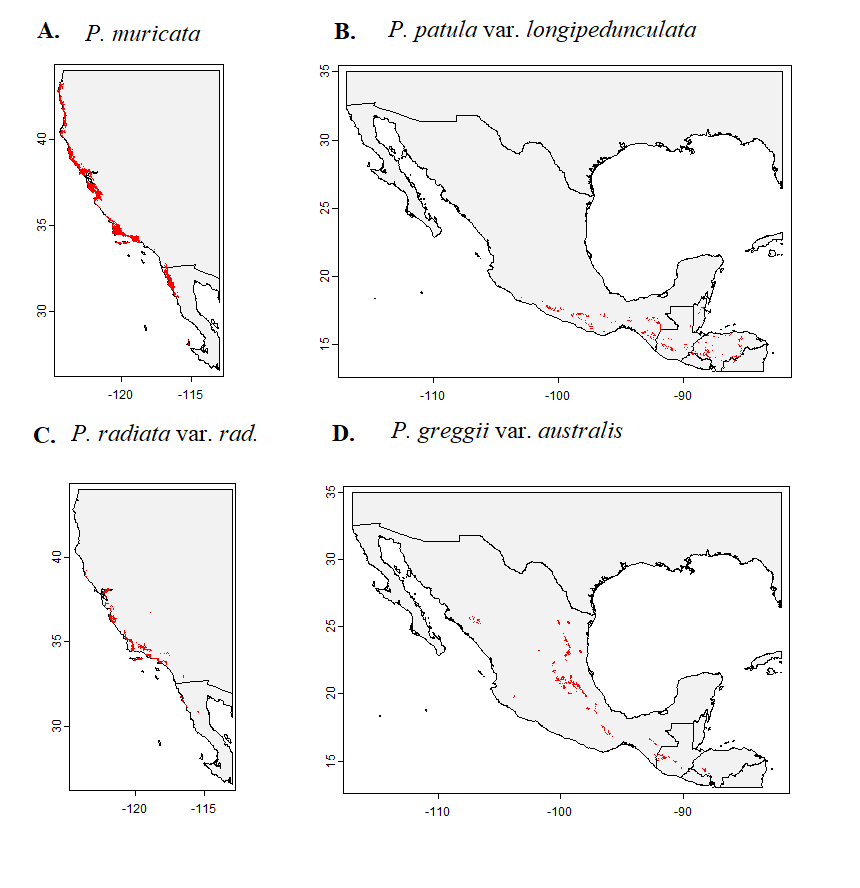


**Figure S4**. Maps of potential distribution projected on the geography using ellipsoids for representative species of the genus *Pinus* (Pinaceae) in the United States, Mexico and Central America.
