# Supplementary material for: A combination of stabilizing selection and random walk are associated with phylogenetic signal in hard pines
Source: Ann Bot. 2025 Jul 16;136(4):821–36. doi: 10.1093/aob/mcaf147 (PMC12464948; doi:10.1093/aob/mcaf147)
Supplement: mcaf147_Supplementary_Data [file mcaf147_supplementary_data.zip › Supp_Tab_JCN_DSG.docx]

**A combination of stabilizing selection and random walk are associated with phylogenetic signal in hard pines**

Jorge Cruz-Nicolás and David S. Gernandt

**Supplementary Tables**

**Table S1.** Collection information for material included in this study. Herbarium codes: MEXU = Herbario Nacional de México, OSC = Oregon State University.

| **Species** | **ID** | **Sample** | **Collected_by** | **Specimen_voucher** | **Subsection** | **GP** | **Description** | **Longitude** | **Latitude** | **Elevation (m asl)** | **Tisssue** | **Location** |
| --- | --- | --- | --- | --- | --- | --- | --- | --- | --- | --- | --- | --- |
| *Pinus contorta* Douglas ex Loudon | contDSG973 | DSG973 | David S. Gernandt | MEXU | Contortae | GP04 | myReads HiSeq 4000, PE 150 | -124.06583 | 44.60556 | 12 | Genomic, diploid from needle | USA: Oregon, Lincoln, south of Newport, South Bay |
| *P. attenuata* Lemmon | atteDSG980 | DSG980 | David S. Gernandt | MEXU | Attenuatae | GP09 | myReads NovaSeq S4, PE150, per Gb (120-140) | -116.60167 | 31.93972 | 814 | Genomic, diploid from needle | Mexico: Baja California, gravel road north of Ensenada |
| *P. radiata* D. Don var. *radiata* | radiDB2241 | DB2241 | B. Bartholomew | MEXU | Attenuatae | GP0102 | High-thoughput sequencing {Illumina, estimate for 1 HiSeq 2500 PE 2X100 lane} | -122.29000 | 37.11000 | 142 | Genomic, haploid from megagametophyte | USA: California, Santa Cruz Co., South of Anyo Nuevo Point |
| *P. radiata* D. Don var. *radiata* | DSG1488_ra | DSG1488 | David S. Gernandt | MEXU | Attenuatae | GP04 | myReads HiSeq 4000, PE 150 | -121.11826 | 35.59969 | 18 | Genomic, diploid from needle | USA: California, Sn Luis Obispo, North of Cambria along San Simeon-Monterey Creek Rd. |
| *P. radiata* var. *binata* (Engelm.) | radiDOB104 | DOB1045 | Dylan O. Burge | MEXU | Attenuatae | GP0102 | High-thoughput sequencing {Illumina, estimate for 1 HiSeq 2500 PE 2X100 lane} | -115.21500 | 28.31800 | 586 | Genomic, diploid from needle | Mexico: Baja California, Ensenada, North end of Cedros Island |
| *P. muricata* D. Don | muriDSG110 | DG1102 | David S. Gernandt | MEXU | Attenuatae | GP09 | myReads NovaSeq S4, PE150, per Gb (120-140) | -122.81472 | 38.09444 | 107 | Genomic, haploid from megagametophyte | USA: California, Point Reyes |
| *P. taeda* L. | taed01s3_U | TAEDA01S3 | David Johnson | MEXU | Australes | GP04 | myReads HiSeq 4000, PE 150 | -75.97806 | 36.85278 | 14 | Genomic, haploid from seed | USA: Virginia, Virginia Beach, Pembrooke Rd |
| *P. taeda* L. | taedDOB129 | DOB1291 | Dylan Orion Burge | MEXU | Australes | GP04 | myReads HiSeq 4000, PE 150 | -78.95000 | 36.01667 | 119 | Genomic, diploid from needle | USA: North Carolina, Duke University Campus |
| *P. taeda* L. | taedGP04_S | TAEDAGP04 | David Johnson | MEXU | Australes | GP04 | myReads HiSeq 4000, PE 150 | -75.97806 | 36.85278 |  | Genomic, haploid from megagametophyte | USA: Virginia, Virginia Beach, Pembrooke Rd |
| *P. rigida* Mill. | rigiDGOLD3 | DGOLD3891 | Doug Goldman | MEXU | Australes | GP0102 | High-thoughput sequencing {Illumina, estimate for 1 HiSeq 2500 PE 2X100 lane} | -70.35000 | 43.87700 | 76 | Genomic, diploid from needle | USA: Maine, Cumberland, Gray Township |
| *P. rigida* Mill. | rigiDG3895 | DGOLD3895 | Doug Goldman | MEXU | Australes | GP0102 | High-thoughput sequencing {Illumina, estimate for 1 HiSeq 2500 PE 2X100 lane} | -71.16420 | 42.46860 | 37 | Genomic, diploid from needle | USA: Massachussetts, Middlesex, Horn Pond |
| *P. rigida* Mill. | rigiRBRIG_ | RBRIG | Richard Bond | MEXU | Australes | GP0102 | High-thoughput sequencing {Illumina, estimate for 1 HiSeq 2500 PE 2X100 lane} | -83.80000 | 35.67600 | 372 | Genomic, diploid from needle | USA: Tennessee, Blount Co., Childhowee Mountains, between Walland and Childhowee |
| *P. serotina* Michx. | seroDOB129 | DOB1293 | Dylan O. Burge | MEXU | Australes | GP0102 | High-thoughput sequencing {Illumina, estimate for 1 HiSeq 2500 PE 2X100 lane} | -77.05600 | 34.91500 | 12 | Genomic, haploid from megagametophyte | USA: North Carolina, Carteret Co., Croatan Game Land |
| *P. chihuahuana* Engelm. | chihDG1066 | DSG1066 | David S. Gernandt | MEXU | Oocarpae | GP0102 | High-thoughput sequencing {Illumina, estimate for 1 HiSeq 2500 PE 2X100 lane} | -108.85059 | 28.38467 | 1605 | Genomic, diploid from needle | Mexico: Sonora, Yecora, Highway 16 east of Yecora |
| *P. chihuahuana* Engelm. | chihDG1588 | DSG1588 | David S. Gernandt | MEXU | Oocarpae | GP09 | myReads NovaSeq S4, PE150, per Gb (120-140) | -107.93065 | 29.13797 | 2183 | Genomic, diploid from needle | Mexico: Chihuahua, Temosachic, East of Madera along Highway 11 |
| *P. chihuahuana* Engelm. | chihDG786_ | DSG786 | David S. Gernandt | MEXU | Oocarpae | GP0102 | High-thoughput sequencing {Illumina, estimate for 1 HiSeq 2500 PE 2X100 lane} | -110.71028 | 32.37194 | 2033 | Genomic, diploid from needle | USA: Arizona, Pima, Catalina Hwy to Mt. Lemmon, near Windy Point Vista |
| *P. cubensis* Griseb. | cubeALCUBE | AL-CUBE01 | Tom Parchman | MEXU | Australes | GP0102 | High-thoughput sequencing {Illumina, estimate for 1 HiSeq 2500 PE 2X100 lane} | -75.79542 | 20.48978 | 700 | Genomic, haploid from megagametophyte | Cuba: Holguin Province, Estacion Pinares de Mayari, Sierra de Nipe |
| *P. echinata* Mill. | echiDOB129 | DOB1292 | Dylan O. Burge | MEXU | Australes | GP0102 | High-thoughput sequencing {Illumina, estimate for 1 HiSeq 2500 PE 2X100 lane} | -78.94177 | 36.00270 | 118 | Genomic, diploid from needle | USA: North Carolina, Durham Co., Duke University |
| *P. elliottii* Engelm. var. *densa* Little & K. W. Dorman | elliDG100 | DSG1002 | David S. Gernandt | MEXU | Australes | GP09 | myReads HiSeq 4000, PE 150 | -81.21889 | 27.80278 | 15 | Genomic, diploid from needle | USA: Florida, River Ranch Blvd off State Road 60 |
| *P. elliottii* Engelm. var. *densa* Little & K. W. Dorman | elliELLI05 | AMW986-ELLI05 | Ann Willyard | OSC | Australes | GP0102 | High-thoughput sequencing {Illumina, estimate for 1 HiSeq 2500 PE 2X100 lane} | -81.36200 | 24.67000 | 1 | Genomic, diploid from needle | USA: Florida, Monroe, Big Pine Key |

**Table S1.** Collection information for material included in this study. Herbarium codes: MEXU = Herbario Nacional de México, OSC = Oregon State University.

(continued…)

| **Species** | **ID** | **Sample** | **Collected_by** | **Specimen_voucher** | **Subsection** | **GP** | **Description** | **Longitude** | **Latitude** | **Elevation (m asl)** | **Tisssue** | **Location** |
| --- | --- | --- | --- | --- | --- | --- | --- | --- | --- | --- | --- | --- |
| *P. georginae* Pérez de la Rosa | SHL08_geor | SHL08 | Sergio Hernandez Leon | MEXU | Oocarpae | GP0102 | High-thoughput sequencing {Illumina, estimate for 1 HiSeq 2500 PE 2X100 lane} | -104.75517 | 20.45789 | 1397 | Genomic, diploid from needle | Mexico: Jalisco, Mascota, Near Talpa and Mascota |
| *P. georginae* Pérez de la Rosa | SHL09_geor | SHL09 | Sergio Hernandez Leon | MEXU | Oocarpae | GP0102 | High-thoughput sequencing {Illumina, estimate for 1 HiSeq 2500 PE 2X100 lane} | -104.75581 | 20.45683 | 1413 | Genomic, diploid from needle | Mexico: Jalisco, Mascota, Near Talpa and Mascota |
| *P. georginae* Pérez de la Rosa | DSG1467_ge | DSG1467 | David S. Gernandt | MEXU | Oocarpae | GP04 | myReads HiSeq 4000, PE 150 | -104.76507 | 20.47887 | 1507 | Genomic, diploid from needle | Mexico: Jalisco, Mascota, East of Mascota along highway 70 |
| *P. glabra* Walter | glabJS1948 | JS1948 | John Schenk | OSC | Australes | GP0102 | High-thoughput sequencing {Illumina, estimate for 1 HiSeq 2500 PE 2X100 lane} | -84.26245 | 30.49872 | 51 | Genomic, diploid from needle | USA: Florida, Leon Co., Timberlane Ravine Park |
| *P. glabra* Walter | glabJS1952 | JS1952 | John Schenk | OSC | Australes | GP0102 | High-thoughput sequencing {Illumina, estimate for 1 HiSeq 2500 PE 2X100 lane} | -84.26245 | 30.49872 | 77 | Genomic, diploid from needle | USA: Florida, Leon Co., Timberlane Ravine Park |
| *P. greggii* var. *greggii* Engelm. ex Parl. | gregDG1308 | DSG1308 | David S. Gernandt | MEXU | Oocarpae | GP0102 | High-thoughput sequencing {Illumina, estimate for 1 HiSeq 2500 PE 2X100 lane} | -99.17010 | 21.08147 | 1606 | Genomic, haploid from megagametophyte | Mexico: Hidalgo, Jacala de Ledezma, Laguna Seca |
| *P. greggii* var. *greggii* Engelm. ex Parl. | gregDG1313 | DSG1313 | David S. Gernandt | MEXU | Oocarpae | GP0102 | High-thoughput sequencing {Illumina, estimate for 1 HiSeq 2500 PE 2X100 lane} | -98.24750 | 20.39763 | 1628 | Genomic, diploid from needle | Mexico: Hidalgo, San Bartolo Tutotepec, La Cumbre de Muridores |
| *P. greggii* var. *greggii* Engelm. ex Parl. | gregDG1325 | DSG1325 | David S. Gernandt | MEXU | Oocarpae | GP0102 | High-thoughput sequencing {Illumina, estimate for 1 HiSeq 2500 PE 2X100 lane} | -98.69858 | 20.79886 | 1800 | Genomic, diploid from needle | Mexico: Hidalgo, Xochicoatlan, Jalamelco, near Molango |
| *P. greggii* var. *australis* Donahue & López | gregDG1349 | DSG1349 | David S. Gernandt | MEXU | Oocarpae | GP0102 | High-thoughput sequencing {Illumina, estimate for 1 HiSeq 2500 PE 2X100 lane} | -100.89968 | 25.36691 | 2378 | Genomic, diploid from needle | Mexico: Coahuila, Arteaga, near El Diamante |
| *P. greggii* var. *australis* Donahue & López | gregDG1365 | DSG1365 | David S. Gernandt | MEXU | Oocarpae | GP0102 | High-thoughput sequencing {Illumina, estimate for 1 HiSeq 2500 PE 2X100 lane} | -100.21371 | 24.89766 | 2597 | Genomic, diploid from needle | Mexico: Nuevo Leon, Galeana, Cerro Potosi |
| *P. greggii* var. *australis* Donahue & López | gregDG1380 | DSG1380 | David S. Gernandt | MEXU | Oocarpae | GP0102 | High-thoughput sequencing {Illumina, estimate for 1 HiSeq 2500 PE 2X100 lane} | -99.68769 | 21.13327 | 2935 | Genomic, haploid from megagametophyte | Mexico: Queretaro, Pinal de Amoles, Cerro de la Pinguica |
| *P. herrerae* Martínez | herrDG1062 | DSG1062 | David S. Gernandt | MEXU | Oocarpae | GP0102 | High-thoughput sequencing {Illumina, estimate for 1 HiSeq 2500 PE 2X100 lane} | -108.87030 | 28.37963 | 1762 | Genomic, diploid from needle | Mexico: Sonora, Yecora, east of Yecora |
| *P. herrerae* Martínez | herrDG634_ | DSG634 | David S. Gernandt | MEXU | Oocarpae | GP0102 | High-thoughput sequencing {Illumina, estimate for 1 HiSeq 2500 PE 2X100 lane} | -103.05417 | 18.81972 | 2184 | Genomic, diploid from needle | México: Michoacán, Coalcomán |
| *P. herrerae* Martínez | herrJP1993 | JAPR1993 | Jorge A. Pérez de la Rosa | MEXU | Oocarpae | GP09 | myReads NovaSeq S4, PE150, per Gb (120-140) | -96.49897 | 16.15928 | 2451 | Genomic, diploid from needle | Mexico: Oaxaca, Miahuatlan, San Mateo Rio Hondo, San Jose del Pacifico |
| *P. jaliscana* Pérez de la Rosa | jaliDG456_ | DSG456 | Sergio Hernández León | MEXU | Oocarpae | GP0102 | High-thoughput sequencing {Illumina, estimate for 1 HiSeq 2500 PE 2X100 lane} | -105.25972 | 20.35722 | 1105 | Genomic, haploid from megagametophyte | Mexico: Jalisco, Cabos Corrientes, El Tuito, road to minas de Zimapan |
| *P. jaliscana* Pérez de la Rosa | jaliSHL11_ | SHL11 | Sergio Hernández León | MEXU | Oocarpae | GP0102 | High-thoughput sequencing {Illumina, estimate for 1 HiSeq 2500 PE 2X100 lane} | -104.89147 | 20.73647 | 1238 | Genomic, diploid from needle | Mexico: Jalisco, Del Oeste, La Estancia |
| *P. lawsonii* Roezl ex Gordon | lawsDG1723 | DSG1723 | David S. Gernandt | MEXU | Oocarpae | GP09 | myReads NovaSeq S4, PE150, per Gb (120-140) | -104.89147 | 20.73647 | 1238 | Genomic, diploid from needle | Mexico: Jalisco, Del Oeste, La Estancia |
| *P. lawsonii* Roezl ex Gordon | lawsDG1726 | DSG1726 | David S. Gernandt | MEXU | Oocarpae | GP09 | myReads NovaSeq S4, PE150, per Gb (120-140) | -99.94428 | 18.82516 | 2186 | Genomic, diploid from needle | Mexico: Mexico, Sultepec, South of Sultepec along Hwy 10 |
| *P. lawsonii* Roezl ex Gordon | lawsJP1842 | JAPR1842 | David S. Gernandt | MEXU | Oocarpae | GP09 | myReads NovaSeq S4, PE150, per Gb (120-140) | -101.96700 | 19.50000 | 2076 | Genomic, diploid from needle | Mexico: Michoacan, Ziracuaretiro, Patzcuaro-Uruapan |
| *P. leiophylla* Schiede ex Schltdl. & Cham. | leioDFM804 | DSFM804 | Dante S. Figueroa M. | MEXU | Oocarpae | GP09 | myReads NovaSeq S4, PE150, per Gb (120-140) | -103.72580 | 19.98132 | 2336 | Genomic, diploid from needle | Mexico: Jalisco, Tapalpa, Camino Tapalpa-Ferrería de Tula |
| *P. leiophylla* Schiede ex Schltdl. & Cham. | leioDG611_ | DSG611 | David S. Gernandt | MEXU | Oocarpae | GP09 | myReads NovaSeq S4, PE150, per Gb (120-140) | -99.22972 | 20.81000 | 2527 | Genomic, diploid from needle | México: Hidalgo, Zimapan, gravel road to Puerto de Piedra |
| *P. leiophylla* Schiede ex Schltdl. & Cham. | leioDG767_ | DSG767 | David S. Gernandt | MEXU | Oocarpae | GP0102 | High-thoughput sequencing {Illumina, estimate for 1 HiSeq 2500 PE 2X100 lane} | -99.54472 | 18.89139 | 2040 | Mexico: Mexico, , Pachuquilla, near Malinalco | Genomic, diploid from needle |
| *P. patula* var. *longipedunculata* Loock | longDGIX4_ | DSG1397-pa4 | David S. Gernandt | MEXU | Oocarpae | GP0102 | High-thoughput sequencing {Illumina, estimate for 1 HiSeq 2500 PE 2X100 lane} | -96.44802 | 17.37530 | 3050 | Genomic, diploid from needle | Mexico: Oaxaca, Ixtlan de Juarez, Cerro de los Pozuelos |
| *P. patula* var. *longipedunculata* Loock | longDGIX5_ | DSG1307-pa5 | David S. Gernandt | MEXU | Oocarpae | GP0102 | High-thoughput sequencing {Illumina, estimate for 1 HiSeq 2500 PE 2X100 lane} | -96.44802 | 17.37530 | 3050 | Genomic, diploid from needle | Mexico: Oaxaca, Ixtlan de Juarez, Cerro de los Pozuelos |
| *P. lumholtzii* B. L. Rob & Fernald | lumhDFM825 | DSFM825 | Dante S. Figueroa M. | MEXU | Oocarpae | GP09 | myReads NovaSeq S4, PE150, per Gb (120-140) | -104.22071 | 20.51935 | 1633 | Genomic, diploid from needle | Mexico: Jalisco, Ameca, Rancho el Devisadero |
| *P. lumholtzii* B. L. Rob & Fernald | lumhDG1079 | DSG1079 | David S. Gernandt | MEXU | Oocarpae | GP0102 | myReads NovaSeq S4, PE150, per Gb (120-140) | -108.03252 | 27.28608 | 1936 | Genomic, diploid from needle | Mexico: Chihuahua, Urique, Between Bahuichivo and Mirador Cerro Gallego |

**Table S1.** Collection information for material included in this study. Herbarium codes: MEXU = Herbario Nacional de México, OSC = Oregon State University.

(continued…)

| **Species** | **ID** | **Sample** | **Collected_by** | **Specimen_voucher** | **Subsection** | **GP** | **Description** | **Longitude** | **Latitude** | **Elevation (m asl)** | **Tisssue** | **Location** |
| --- | --- | --- | --- | --- | --- | --- | --- | --- | --- | --- | --- | --- |
| *P. lumholtzii* B. L. Rob & Fernald | lumhDG937_ | DSG937 | David S. Gernandt | MEXU | Oocarpae | GP04 | myReads HiSeq 4000, PE 150 | -105.83800 | 23.62300 | 2213 | Genomic, diploid from needle | Mexico: Durango, El Salto, Highway 40 south of El Salto |
| *P. luzmariae* Pérez de la Rosa | luzmDG1167 | DSG1167 | David S. Gernandt | MEXU | Oocarpae | GP0102 | High-thoughput sequencing {Illumina, estimate for 1 HiSeq 2500 PE 2X100 lane} | -104.59100 | 20.37296 | 1934 | Genomic, diploid from needle | Mexico: Jalisco, Atenguillo, La Campana |
| *P. luzmariae* Pérez de la Rosa | luzmDG1476 | DSG1476 | David S. Gernandt | MEXU | Oocarpae | GP04 | myReads HiSeq 4000, PE 150 | -104.49555 | 20.25621 | 1736 | Genomic, diploid from needle | Mexico: Jalisco, Atenguillo |
| *P. luzmariae* Pérez de la Rosa | luzmDSFM185 | DSFM185 | Dante S. Figueroa M. | MEXU | Oocarpae | GP09 | myReads NovaSeq S4, PE150, per Gb (120-140) | -104.41739 | 22.36550 | 1940 | Genomic, diploid from needle | México: Nayarit, San Andrés cohemiata |
| *P. occidentalis* Sw. | occiLJB2_S | LJB2 | Lev Jardon Barbolla | MEXU | Australes | GP0102 | High-thoughput sequencing {Illumina, estimate for 1 HiSeq 2500 PE 2X100 lane} | -70.69400 | 18.84200 | 1833 | Genomic, diploid from needle | Dominican Republic: Agua Blanca |
| *P. oocarpa* Schiede ex Schltdl. | oocaDFM494 | DSFM494 | Dante S. Figueroa M. | MEXU | Oocarpae | GP09 | myReads NovaSeq S4, PE150, per Gb (120-140) | -103.46861 | 20.93806 | 1597 | Genomic, diploid from needle | Mexico: Jalisco, Zapopan, 3.5 km west of Lomas del Calvario |
| *P. oocarpa* Schiede ex Schltdl. | oocaDFM824 | DSFM824 | Dante S. Figueroa M. | MEXU | Oocarpae | GP09 | myReads NovaSeq S4, PE150, per Gb (120-140) | -104.22071 | 20.51935 | 1634 | Genomic, diploid from needle | Mexico: Jalisco, Ameca, Rancho el devisadero |
| *P. oocarpa* Schiede ex Schltdl. | oocaDG726_ | DSG726 | David S. Gernandt | MEXU | Oocarpae | GP02 | High-thoughput sequencing {Illumina, estimate for 1 HiSeq 2500 PE 2X100 lane} | -103.61944 | 20.72667 | 1471 | Genomic, haploid from megagametophyte | Mexico: Jalisco, Guadalajara, Tala, west of Guadalajara along road |
| *P. palustris* Mill. | paluDGOLD3 | DGOLD3915 | Doug Goldman | MEXU | Australes | GP0102 | High-thoughput sequencing {Illumina, estimate for 1 HiSeq 2500 PE 2X100 lane} | -84.97764 | 29.99731 | 9 | Genomic, diploid from needle | USA: Florida, Franklin Co., Apalachicola National Forest |
| *P. patula* var. *patula* Schiede ex Schltdl. & Cham. | patuAOM10_ | AOM10 | Alfredo Ortiz Martinez | MEXU | Oocarpae | GP05 | High-thoughput sequencing {Illumina, estimate for 1 HiSeq 2500 PE 2X100 lane} | -99.54167 | 19.72722 | 2799 | Genomic, diploid from needle | Mexico: Mexico, Villa del Carbon |
| *P. patula* var. *patula* Schiede ex Schltdl. & Cham. | patuDG1330 | DSG1330 | David S. Gernandt | MEXU | Oocarpae | GP0102 | High-thoughput sequencing {Illumina, estimate for 1 HiSeq 2500 PE 2X100 lane} | -98.66852 | 20.70527 | 1988 | Genomic, diploid from needle | Mexico: Hidalgo, Zacualtipan de Angeles, road between Zacualtipan and Tianguistango |
| *P. patula* var. *patula* Schiede ex Schltdl. & Cham. | patuDRD32_ | DRD32 | Diana Ramos Dorantes | MEXU | Oocarpae | GP0102 | High-thoughput sequencing {Illumina, estimate for 1 HiSeq 2500 PE 2X100 lane} | -97.48350 | 19.88774 | 1696 | Genomic, diploid from needle | Mexico: Puebla, Tlatlauquitepec, road Progreso-Tlatlauquitepec |
| *P. praetermissa* Styles & McVaugh | praeDFM504 | DSFM504 | Dante S. Figueroa M. | MEXU | Oocarpae | GP09 | myReads NovaSeq S4, PE150, per Gb (120-140) | -104.04972 | 20.98778 | 1447 | Genomic, diploid from needle | Mexico: Jalisco, Hostotipaquillo, cerro junto al Camino a Hostotipaquillo |
| *P. praetermissa* Styles & McVaugh | praeDG454_ | DSG454 | David S. Gernandt | MEXU | Oocarpae | GP09 | myReads NovaSeq S4, PE150, per Gb (120-140) | -104.04972 | 20.98833 | 1437 | Genomic, diploid from needle | Mexico: Jalisco, Magdalena, just north of hwy 15 |
| *P. praetermissa* Styles & McVaugh | praeDSFM18 | DSFM188 | Dante S. Figueroa M. | MEXU | Oocarpae | GP09 | myReads NovaSeq S4, PE150, per Gb (120-140) | -100.72722 | 22.16917 | 1213 | Genomic, diploid from needle | Mexico: Nayarit, El Maguey, Km 14.2 después de la "Mesa del Nayar" |
| *P. pringlei* Shaw | prinDG1721 | DSG1721 | David S. Gernandt | MEXU | Oocarpae | GP09 | myReads NovaSeq S4, PE150, per Gb (120-140) | -99.94430 | 18.82516 | 2185 | Genomic, diploid from needle | Mexico: Mexico, Sultepec, South of Sultepec along Hwy 10 |
| *P. pringlei* Shaw | prinDG1725 | DSG1725 | David S. Gernandt | MEXU | Oocarpae | GP09 | myReads NovaSeq S4, PE150, per Gb (120-140) | -99.93296 | 18.82221 | 2077 | Genomic, diploid from needle | Mexico: Mexico, Sultepec, South of Sultepec along Hwy 10 |
| *P. pringlei* Shaw | prinJP1831 | JPR1831 | Jorge A. Perez de la Rosa | MEXU | Oocarpae | GP02 | High-thoughput sequencing {Illumina, estimate for 1 HiSeq 2500 PE 2X100 lane} | -99.94430 | 18.82516 | 2187 | Genomic, haploid from megagametophyte | Mexico: Estado de Mexico, Sultepec (aproximate) |
| *P. pungens* Lamb | pungRBPUN_ | RBPUN | Richard Bond | MEXU | Australes | GP0102 | High-thoughput sequencing {Illumina, estimate for 1 HiSeq 2500 PE 2X100 lane} | -83.81389 | 35.70500 | 350 | Genomic, haploid from megagametophyte | USA: Tennessee, Blount Co., Childhowee Mountains, Foothills Parkway |
| *P. tecunumanii* Eguiluz & J. P. Perry | tecuDG895_ | DSG895 | David S. Gernandt | MEXU | Oocarpae | GP0102 | High-thoughput sequencing {Illumina, estimate for 1 HiSeq 2500 PE 2X100 lane} | -92.52222 | 16.62167 | 2251 | Genomic, diploid from needle | Mexico: Chiapas, San Cristobal de las Casas, southeast of San Cristobal de las Casas |
| *P. tecunumanii* Eguiluz & J. P. Perry | tecuDG896_ | DSG896 | David S. Gernandt | MEXU | Oocarpae | GP0102 | High-thoughput sequencing {Illumina, estimate for 1 HiSeq 2500 PE 2X100 lane} | -90.35278 | 14.53611 | 1975 | Genomic, diploid from needle | Guatemala: Guatemala, San Jose Pinula |
| *P. teocote* Schiede ex Schtdl. & Cham. | teocDG1403 | DSG1403 | David S. Gernandt | MEXU | Oocarpae | GP0102 | High-thoughput sequencing {Illumina, estimate for 1 HiSeq 2500 PE 2X100 lane} | -99.69379 | 21.15937 | 3074 | Genomic, diploid from needle | Mexico: Queretaro, Pinal de Amoles, Cerro de la Pinguica |
| *P. teocote* Schiede ex Schtdl. & Cham. | teocDG1719 | DSG1719 | David S. Gernandt | MEXU | Oocarpae | GP09 | myReads NovaSeq S4, PE150, per Gb (120-140) | -98.60613 | 20.11076 | 2708 | Genomic, diploid from needle | Mexico: Hidalgo, Epaza, El Guajalote |
| *P. teocote* Schiede ex Schtdl. & Cham. | teocDG540_ | DSG540 | David S. Gernandt | MEXU | Oocarpae | GP0102 | High-thoughput sequencing {Illumina, estimate for 1 HiSeq 2500 PE 2X100 lane} | -99.70500 | 23.60889 | 2718 | Genomic, haploid from megagametophyte | Mexico: Tamaulipas, Miquihuana, road La Pena-El Aserradero, N of Miquihuana |
| *P. vallartensis* Pérez de la Rosa & Gernandt | vallDG1527 | DSG1527 | David S. Gernandt | MEXU | Oocarpae | GP04 | myReads HiSeq 4000, PE 150 | -105.24500 | 20.53200 | 369 | Genomic, diploid from needle | Mexico: Jalisco, Puerto Vallarta |
| *P. vallartensis* Pérez de la Rosa & Gernandt | vallDG1529 | DSG1529 | David S. Gernandt | MEXU | Oocarpae | GP09 | myReads HiSeq 4000, PE 150 | -105.24460 | 20.53090 | 414 | Genomic, haploid from megagametophyte | Mexico: Jalisco, Puerto Vallarta |
| *P. vallartensis* Pérez de la Rosa & Gernandt | vallDG1531 | DSG1531 | David S. Gernandt | MEXU | Oocarpae | GP04 | myReads HiSeq 4000, PE 150 | -105.24500 | 20.53100 | 409 | Genomic, diploid from needle | Mexico: Jalisco, Puerto Vallarta |

**Table S2.** Mean values for variables evaluated in this study.

| **Species** | **Needles**  **per**  **fascicle** | **Needle**  **length**  **(cm)** | **Number**  **of**  **resin**  **canals** | **Cone**  **length**  **(cm)** | **Bark**  **thickness**  **(mm)** | **Nitrogen**  **cg/kg** | **Org**  **carbon**  **dg/kg** | **Cat**  **exchange mmol(c)/kg** | **pH** | **DNA**  **content (pg)** | **bio1** | **bio5** | **bio6** | **bio7** | **bio12** | **bio13** | **bio17** |
| --- | --- | --- | --- | --- | --- | --- | --- | --- | --- | --- | --- | --- | --- | --- | --- | --- | --- |
| *P. coulteri* | 3 | 23.000 | 3 | 25.000 | 65.000 | 363.667 | 337.500 | 192.500 | 6.750 | 30.630 | 12.889 | 29.044 | 5.000 | 285.444 | 640.667 | 117.778 | 19.778 |
| *P. attenuata* | 3 | 12.000 | 3 | 11.500 | 22.500 | 434.400 | 352.800 | 255.600 | 6.620 | 31.000 | 12.138 | 29.206 | -0.100 | 29.306 | 988.750 | 186.063 | 26.563 |
| *P. muricata* | 2 | 11.000 | 3 | 7.000 | 17.500 | 426.778 | 589.600 | 250.133 | 6.313 | 25.370 | 13.869 | 24.697 | 4.356 | 20.341 | 683.406 | 135.156 | 10.125 |
| *P. radiata* | 2 | 12.000 | 2 | 11.000 | 22.500 | 403.000 | 764.333 | 235.667 | 6.133 | 24.250 | 14.056 | 25.333 | 3.822 | 21.511 | 462.778 | 98.000 | 7.778 |
| *P. radiata*  var. *binata* | 2 | 11.500 | 2 | 7.750 | 22.500 | 177.714 | 127.750 | 148.625 | 6.913 | 24.250 | 16.650 | 26.943 | 7.986 | 18.957 | 134.071 | 28.071 | 3.786 |
| *P. glabra* | 2 | 7.000 | 2 | 7.000 | 14.000 | 1614.125 | 597.294 | 202.000 | 5.041 | 20.900 | 18.817 | 32.937 | 3.061 | 29.876 | 1448.010 | 173.295 | 265.200 |
| *P. pungens* | 2 | 6.000 | 2 | 8.000 | 25.000 | 610.320 | 1293.526 | 339.404 | 4.904 | 23.960 | 10.703 | 27.869 | -6.497 | 34.367 | 1201.702 | 120.349 | 254.890 |
| *P. rígida* | 2 | 7.500 | 2 | 7.000 | 22.500 | 793.795 | 1239.737 | 345.632 | 5.011 | 24.150 | 9.510 | 27.741 | -8.501 | 36.242 | 1160.384 | 116.038 | 252.709 |
| *P. serotina* | 3 | 17.500 | 2 | 6.500 | 17.500 | 881.400 | 561.000 | 189.250 | 5.050 | 21.500 | 18.166 | 32.272 | 2.516 | 29.756 | 1308.375 | 169.656 | 234.063 |
| *P. taeda* | 3 | 17.000 | 2 | 9.000 | 22.500 | 356.917 | 598.600 | 188.400 | 5.260 | 22.100 | 16.590 | 32.430 | -0.103 | 32.533 | 1267.815 | 142.444 | 251.919 |
| *P. occidentalis* | 3 | 14.500 | 3 | 6.000 | 30.000 | 489.600 | 598.400 | 283.800 | 5.973 | 22.940 | 21.593 | 29.100 | 13.233 | 15.867 | 1374.267 | 216.067 | 150.333 |
| *P. cubensis* | 3 | 12.000 | 4 | 5.500 | 22.500 | 553.100 | 732.923 | 306.385 | 5.869 | 22.940 | 23.114 | 30.129 | 15.790 | 14.338 | 1590.857 | 243.238 | 184.905 |
| *P. palustris* | 3 | 32.500 | 4 | 20.000 | 27.500 | 937.149 | 532.938 | 176.250 | 5.008 | 24.050 | 18.324 | 32.641 | 2.581 | 30.060 | 1345.741 | 164.741 | 247.252 |
| *P. elliottii* | 3 | 22.500 | 3 | 13.000 | 22.500 | 1396.500 | 682.875 | 215.375 | 5.063 | 22.610 | 20.735 | 32.616 | 6.895 | 25.720 | 1388.534 | 189.909 | 220.534 |
| *P. echinata* | 3 | 9.000 | 3 | 5.500 | 22.500 | 439.900 | 630.933 | 194.533 | 5.213 | 22.750 | 15.527 | 32.248 | -2.019 | 34.267 | 1290.258 | 141.565 | 255.300 |
| *P. patula*  var. *patula* | 3 | 17.000 | 3 | 7.750 | 15.000 | 426.538 | 540.636 | 250.273 | 6.045 | 21.920 | 13.683 | 23.725 | 3.992 | 19.733 | 1296.667 | 288.833 | 84.250 |
| *P. patula* var.  *longipedunculata* | 3 | 19.250 | 3 | 6.750 | 15.000 | 450.000 | 702.000 | 231.000 | 5.180 | 21.920 | 16.463 | 25.550 | 7.700 | 17.850 | 1513.375 | 314.000 | 48.500 |
| *P. greggii*  var. *greggii* | 3 | 9.400 | 3 | 10.500 | 15.000 | 436.923 | 686.100 | 207.000 | 6.880 | 20.680 | 12.830 | 23.530 | 1.620 | 21.910 | 556.900 | 89.900 | 61.600 |
| *P. greggi*  var. *australis* | 3 | 12.500 | 3 | 10.500 | 15.000 | 472.571 | 535.000 | 264.357 | 6.336 | 20.680 | 17.550 | 28.421 | 6.143 | 22.279 | 1315.643 | 293.214 | 88.571 |
| *P. tecunumanii* | 4 | 17.000 | 3 | 5.500 | 22.500 | 428.143 | 507.313 | 304.250 | 6.263 | 20.490 | 19.229 | 27.164 | 11.024 | 16.140 | 1713.746 | 310.218 | 82.127 |
| *P. leiophylla* | 5 | 12.000 | 2 | 4.500 | 27.500 | 369.898 | 481.348 | 240.043 | 5.809 | 24.830 | 14.189 | 25.287 | 2.474 | 22.813 | 943.541 | 199.623 | 35.672 |
| *P. herrerae* | 3 | 10.000 | 2 | 3.500 | 20.000 | 533.867 | 570.900 | 237.150 | 5.730 | 24.600 | 15.770 | 26.750 | 4.225 | 22.525 | 1133.350 | 264.650 | 43.800 |
| *P. chihuahuana* | 3 | 10.000 | 3 | 5.000 | 15.000 | 383.000 | 369.000 | 236.600 | 6.093 | 24.830 | 14.556 | 30.284 | -0.806 | 31.091 | 649.656 | 154.313 | 36.688 |
| *P. lumholtzii* | 3 | 25.000 | 4 | 4.500 | 15.000 | 445.824 | 418.059 | 240.059 | 5.759 | 24.830 | 15.921 | 27.556 | 3.874 | 23.682 | 940.794 | 228.441 | 32.500 |
| *P. pringlei* | 3 | 21.500 | 4 | 6.500 | 15.000 | 289.000 | 382.000 | 213.000 | 6.800 | 22.340 | 17.417 | 26.733 | 8.300 | 18.433 | 1306.667 | 283.333 | 29.167 |
| *P. teocote* | 3 | 12.500 | 3 | 5.000 | 15.000 | 394.000 | 490.214 | 222.357 | 6.329 | 24.600 | 15.425 | 25.963 | 4.563 | 21.400 | 870.625 | 184.938 | 39.438 |

**Table S2.** Mean values for variables evaluated in this study. (continued…)

| **Species** | **Needles**  **per**  **fascicle** | **Needle**  **length**  **(cm)** | **Number**  **of**  **resin**  **canals** | **Cone**  **length**  **(cm)** | **Bark**  **thickness**  **(mm)** | **Nitrogen**  **cg/kg** | **Org**  **carbon**  **dg/kg** | **Cat**  **exchange mmol(c)/kg** | **pH** | **DNA**  **content (pg)** | **bio1** | **bio5** | **bio6** | **bio7** | **bio12** | **bio13** | **bio17** |
| --- | --- | --- | --- | --- | --- | --- | --- | --- | --- | --- | --- | --- | --- | --- | --- | --- | --- |
| *P. lawsonii* | 3 | 16.000 | 4 | 6.500 | 15.000 | 272.500 | 386.000 | 196.500 | 6.650 | 21.740 | 17.225 | 27.450 | 6.675 | 20.775 | 1160.000 | 255.500 | 24.750 |
| *P. oocarpa* | 5 | 23.000 | 5 | 7.400 | 17.500 | 429.339 | 480.646 | 240.583 | 5.731 | 21.740 | 20.406 | 30.393 | 10.006 | 20.387 | 1431.111 | 310.704 | 46.565 |
| *P. jaliscana* | 5 | 15.000 | 5 | 7.250 | 17.500 | 366.000 | 373.200 | 229.200 | 5.700 | 21.780 | 20.620 | 30.660 | 9.360 | 21.300 | 1379.800 | 331.800 | 24.200 |
| *P. vallartensis* | 5 | 18.500 | 5 | 2.750 | 17.500 | 336.200 | 390.800 | 222.800 | 5.780 | 21.780 | 23.080 | 31.820 | 12.100 | 19.720 | 1440.400 | 353.200 | 22.200 |
| *P. praetermissa* | 5 | 13.000 | 2 | 5.000 | 17.500 | 368.571 | 265.000 | 241.500 | 6.150 | 21.780 | 22.922 | 33.322 | 11.156 | 22.167 | 944.556 | 238.444 | 26.444 |
| *P. georginae* | 5 | 12.000 | 5 | 7.000 | 17.500 | 290.429 | 279.143 | 245.429 | 5.871 | 21.780 | 18.286 | 29.214 | 6.814 | 22.400 | 1219.714 | 313.286 | 27.286 |
| *P. luzmariae* | 3 | 23.000 | 5 | 3.750 | 17.500 | 382.636 | 433.250 | 242.625 | 5.706 | 21.740 | 16.496 | 27.563 | 4.967 | 22.596 | 1010.417 | 249.167 | 29.000 |

**Table S3**. Results of general linear models (needles per fascicle and number of resin canals) and linear models for DNA content, morphological and anatomical characters, with climate variables and PGLS.

| **Response: Needles per fascicle** | | |  |  |  | **Response: Number of resin canals** | | |  |  |  |
| --- | --- | --- | --- | --- | --- | --- | --- | --- | --- | --- | --- |
| **Estimate** | **Coefficients** | **Std. Error** | ***z-value*** | ***Pr(>\|z\|)*** |  | **Estimate** | **Coefficients** | **Std. Error** | ***z-value*** | ***Pr(>\|z\|)*** |  |
| Intercept | 0.35873 | 0.51695 | 0.69400 | 0.48800 |  | Intercept | 0.44806 | 0.51957 | 0.86200 | 0.38800 |  |
| Annual mean temp. (bio1) | 0.04709 | 0.02904 | 1.62200 | 0.10500 |  | Annual mean temp. (bio1) | 0.04085 | 0.02930 | 1.39400 | 0.16300 |  |
| Intercept | 0.39535 | 1.00782 | 0.39200 | 0.69500 |  | Intercept | 0.81239 | 1.01286 | 0.80200 | 0.42300 |  |
| Max. temp. of warmest month (bio5) | 0.02688 | 0.03472 | 0.77400 | 0.43900 |  | Max. temp. of warmest month (bio5) | 0.01173 | 0.03504 | 0.33500 | 0.73800 |  |
| Intercept | 1.02273 | 0.14700 | 6.95700 | 0.00000 |  | Intercept | 0.99392 | 0.14919 | 6.66200 | 0.00000 |  |
| Min temp. of coldest month (bio6) | 0.02767 | 0.01930 | 1.43400 | 0.15200 |  | Min temp. of coldest month (bio6) | 0.02929 | 0.01952 | 1.50100 | 0.13300 |  |
| Intercept | 1.55621 | 0.42446 | 3.66600 | 0.00025 |  | Intercept | 1.66169 | 0.43024 | 3.86200 | 0.00011 |  |
| Temp. Annual range (bio7) | -0.01648 | 0.01777 | -0.92800 | 0.35364 |  | Temp. Annual range (bio7) | -0.02188 | 0.01814 | -1.20600 | 0.22775 |  |
| Intercept | 0.83573 | 0.36544 | 2.28700 | 0.02220 |  | Intercept | 0.78301 | 0.37160 | 2.10700 | 0.03510 |  |
| Annual precipitation (bio12) | 0.00029 | 0.00030 | 0.96000 | 0.33700 |  | Annual precipitation (bio12) | 0.00032 | 0.00030 | 1.03900 | 0.29890 |  |
| Intercept | 0.60904 | 0.30376 | 2.00500 | 0.04500 |  | Intercept | 0.56580 | 0.30789 | 1.83800 | 0.06610 |  |
| Precipitation of wettest month (bio13) | **0.00254** | **0.00126** | **2.01900** | **0.04350** |  | Precipitation of wettest month (bio13) | **0.00265** | **0.00127** | **2.07800** | **0.03770** |  |
| Intercept | 1.28062 | 0.13858 | 9.24100 | 0.00000 |  | Intercept | 1.26484 | 0.13981 | 9.04700 | <2e-16 |  |
| Precipitation of driest quarter (bio17) | -0.00120 | 0.00110 | -1.08500 | 0.27800 |  | Precipitation of driest quarter (bio17) | -0.00124 | 0.00112 | -1.11100 | 0.26700 |  |
| Intercept | 1.34554 | 0.20626 | 6.52300 | 0.00000 |  | Intercept | 1.30873 | 0.20717 | 6.31700 | 0.00000 |  |
| Nitrogen in soil | -0.00035 | 0.00037 | -0.94800 | 0.34300 |  | Nitrogen in soil | -0.00032 | 0.00037 | -0.85500 | 0.39200 |  |
| Intercept | 1.53081 | 0.26275 | 5.82600 | 0.00000 |  | Intercept | 1.45743 | 0.26376 | 5.52600 | 0.00000 |  |
| Soil organic carbon | -0.00068 | 0.00047 | -1.44100 | 0.15000 |  | Soil organic carbon | -0.00058 | 0.00047 | -1.22800 | 0.21900 |  |
| Intercept | 1.21897 | 0.55576 | 2.19300 | 0.02830 |  | Intercept | 1.22789 | 0.56166 | 2.18600 | 0.02880 |  |
| Cation exchange capacity | -0.00021 | 0.00231 | -0.09100 | 0.92730 |  | Cation exchange capacity | -0.00033 | 0.00233 | -0.14200 | 0.88720 |  |
| Intercept | 0.98923 | 0.99071 | 0.99900 | 0.31800 |  | Intercept | 0.74057 | 1.00259 | 0.73900 | 0.46000 |  |
| pH in soil | 0.00307 | 0.01682 | 0.18300 | 0.85500 |  | pH in soil | 0.00698 | 0.01699 | 0.41100 | 0.68100 |  |
| **Response: Needles per fascicle** | | |  |  |  | **Response: Number of resin canals PGLS** | | | |  |  |
| **Estimate** | **Coefficients** | **Std. Error** | ***t-value*** | ***p-value*** | ***λ*** | **Estimate** | **Coefficients** | **Std. Error** | ***t-value*** | ***p-value*** | ***λ*** |
| Intercept | 2.86485 | 0.44830 | 6.39045 | 0.00000 |  | Intercept | 2.01034 | 0.65263 | 3.08036 | 0.00430 |  |
| Precipitation of wettest month (bio13) | 0.00103 | 0.00094 | 1.09600 | 0.28150 | 1.14347 | Precipitation of wettest month (bio13) | **0.00756** | **0.00146** | **5.17200** | **0.00000** | **1.01866** |

**Table S3**. Results of general linear models (needles per fascicle and number of resin canals) and linear models for DNA content, morphological and anatomical characters, with climate variables and PGLS. (continued…)

| **Response: Needle length** |  |  |  |  |  | **Response: Cone length** | | |  |  |  |
| --- | --- | --- | --- | --- | --- | --- | --- | --- | --- | --- | --- |
| **Estimate** | **Coefficients** | **Std. Error** | ***t-value*** | ***p-value*** | ***r2 adj.*** | **Estimate** | **Coefficients** | **Std. Error** | ***t-value*** | ***p-value*** | ***r2 adj.*** |
| Intercept | 3.72420 | 4.98700 | 0.74700 | 0.46100 |  | Intercept | 9.29970 | 3.00860 | 3.09100 | 0.00428 |  |
| Annual mean temp. (bio1) | **0.66340** | **0.28870** | **2.29800** | **0.02870** | **0.12130** | Annual mean temp. (bio1) | -0.11660 | 0.17420 | -0.67000 | 0.50829 | -0.01812 |
| Intercept | 3.65590 | 10.61720 | 0.34400 | 0.73300 |  | Intercept | 2.40550 | 5.99540 | 0.40100 | 0.69100 |  |
| Max. temp. of warmest month (bio5) | 0.39420 | 0.36850 | 1.07000 | 0.29300 | 0.00463 | Max. temp. of warmest month (bio5) | 0.17160 | 0.20810 | 0.82500 | 0.41600 | -0.01043 |
| Intercept | 13.11250 | 1.37560 | 9.53200 | 0.00000 |  | Intercept | 7.96610 | 0.80080 | 9.94700 | 0.00000 |  |
| Min temp. of coldest month (bio6) | 0.37560 | 0.19150 | 1.96100 | 0.05920 | 0.08411 | Min temp. of coldest month (bio6) | -0.13050 | 0.11150 | -1.17100 | 0.25100 | 0.01180 |
| Intercept | 20.13800 | 4.44800 | 4.52700 | 0.00009 |  | Intercept | 3.71990 | 2.46060 | 1.51200 | 0.14100 |  |
| Temp. Annual range (bio7) | -0.21800 | 0.18200 | -1.19800 | 0.24000 | 0.01383 | Temp. Annual range (bio7) | 0.15170 | 0.10070 | 1.50700 | 0.14200 | 0.03939 |
| Intercept | 9.07380 | 3.50448 | 2.58900 | 0.01470 |  | Intercept | 7.62150 | 2.06162 | 3.69700 | 0.00087 |  |
| Annual precipitation (bio12) | 0.00516 | 0.00294 | 1.75400 | 0.08960 | 0.06280 | Annual precipitation (bio12) | -0.00026 | 0.00173 | -0.15000 | 0.88156 | -0.03256 |
| Intercept | 9.18292 | 2.79523 | 3.28500 | 0.00260 |  | Intercept | 10.06449 | 1.60233 | 6.28100 | 0.00000 |  |
| Precipitation of wettest month (bio13) | **0.02722** | **0.01233** | **2.20900** | **0.03500** | **0.11120** | Precipitation of wettest month (bio13) | -0.01291 | 0.00707 | -1.82800 | 0.07750 | 0.07021 |
| Intercept | 15.29773 | 1.54129 | 9.92500 | 0.00000 |  | Intercept | 6.19848 | 0.81710 | 7.58600 | 0.00000 |  |
| Precipitation of driest quarter (bio17) | -0.00346 | 0.01133 | -0.30500 | 0.76300 | -0.03014 | Precipitation of driest quarter (bio17) | 0.01145 | 0.00601 | 1.90600 | 0.06630 | 0.07830 |
| Intercept | 14.47000 | 2.11900 | 6.82800 | 0.00000 |  | Intercept | 5.24790 | 1.10534 | 4.74800 | 0.00005 |  |
| Nitrogen in soil | 0.00094 | 0.00353 | 0.26600 | 0.79200 | -0.03089 | Nitrogen in soil | **0.00401** | **0.00184** | **2.17300** | **0.03780** | **0.10720** |
| Intercept | 19.20228 | 2.59300 | 7.40500 | 0.00000 |  | Intercept | 6.16136 | 1.51042 | 4.07900 | 0.00031 |  |
| Soil organic carbon | -0.00772 | 0.00434 | -1.77800 | 0.08550 | 0.06522 | Soil organic carbon | 0.00212 | 0.00253 | 0.83700 | 0.40919 | 0.00000 |
| Intercept | 25.40235 | 5.68311 | 4.47000 | 0.00010 |  | Intercept | 11.32543 | 3.28233 | 3.45000 | 0.00168 |  |
| Cation exchange capacity | -0.04400 | 0.02356 | -1.86700 | 0.07163 | 0.07428 | Cation exchange capacity | -0.01685 | 0.01361 | -1.23800 | 0.22515 | 0.01693 |
| Intercept | 22.85930 | 10.60340 | 2.15600 | 0.03920 |  | Intercept | 11.42151 | 5.95075 | 1.91900 | 0.06450 |  |
| pH in soil | -0.13510 | 0.18040 | -0.74900 | 0.45970 | 0.00000 | pH in soil | -0.07003 | 0.10123 | -0.69200 | 0.49440 | 0.00000 |
| **Response: Needle length PGLS** |  |  |  |  |  | **Response: Cone length PGLS** | | |  |  |  |
| **Estimate** | **Coefficients** | **Std. Error** | ***t-value*** | ***p-value*** | ***λ*** | **Estimate** | **Coefficients** | **Std. Error** | ***t-value*** | ***p-value*** | ***λ*** |
| Intercept | 1.89740 | 0.31612 | 6.00217 | 0.00000 |  | Intercept | 1.66992 | 0.21926 | 7.61601 | 0.00000 |  |
| Annual mean temp. (bio1) | **0.04435** | **0.01863** | **2.37992** | **0.02370** | **-0.02463** | Nitrogen in soil | 0.00026 | 0.00025 | 1.01087 | 0.31990 | 0.412428 |
| Intercept | 2.24679 | 0.14973 | 15.00610 | 0.00000 |  |  |  |  |  |  |  |
| Precipitation of wettest month (bio13) | **0.00188** | **0.00076** | **2.48081** | **0.01870** | **-0.07840** |  |  |  |  |  |  |

**Table S3**. Results of general linear models (needles per fascicle and number of resin canals) and linear models for DNA content, morphological and anatomical characters, with climate variables and PGLS. (continued…)

| **Response: Bark thickness** |  |  |  |  |  | **Response: DNA content** |  |  |  |  |  |
| --- | --- | --- | --- | --- | --- | --- | --- | --- | --- | --- | --- |
| **Estimate** | **Coefficients** | **Std. Error** | ***t-value*** | ***p-value*** | ***r2 adj.*** | **Estimate** | **Coefficients** | **Std. Error** | ***t-value*** | ***p-value*** | ***r2 adj.*** |
| Intercept | 19.76141 | 3.99033 | 4.95200 | 0.00003 |  | Intercept | 27.79842 | 1.63748 | 16.97600 | 0.00000 |  |
| Annual mean temp. (bio1) | -0.02005 | 0.23103 | -0.08700 | 0.93100 | -0.03307 | Annual mean temp. (bio1) | **-0.28184** | **0.09481** | **-2.97300** | **0.00577** | **0.20180** |
| Intercept | 12.61410 | 7.88470 | 1.60000 | 0.12000 |  | Intercept | 26.10240 | 3.68390 | 7.08600 | 0.00000 |  |
| Max. temp. of warmest month (bio5) | 0.23740 | 0.27360 | 0.86800 | 0.39200 | -0.00804 | Max. temp. of warmest month (bio5) | -0.10730 | 0.12790 | -0.83900 | 0.40800 | -0.00963 |
| Intercept | 19.79211 | 1.07374 | 18.43300 | <2e-16 |  | Intercept | 23.70855 | 0.46926 | 50.52400 | <2e-16 |  |
| Min temp. of coldest month (bio6) | -0.07536 | 0.14949 | -0.50400 | 0.61800 | -0.02465 | Min temp. of coldest month (bio6) | **-0.13895** | **0.06533** | **-2.12700** | **0.04180** | **0.10210** |
| Intercept | 16.53060 | 3.31600 | 4.98500 | 0.00002 |  | Intercept | 20.88435 | 1.51628 | 13.77300 | 0.00000 |  |
| Temp. Annual range (bio7) | 0.12170 | 0.13570 | 0.89700 | 0.37700 | -0.00635 | Temp. Annual range (bio7) | 0.09013 | 0.06204 | 1.45300 | 0.15700 | 0.03458 |
| Intercept | 18.60924 | 2.71111 | 6.86400 | 0.00000 |  | Intercept | 25.80182 | 1.15188 | 22.40000 | <2e-16 |  |
| Annual precipitation (bio12) | 0.00071 | 0.00228 | 0.31300 | 0.75600 | -0.02997 | Annual precipitation (bio12) | **-0.00244** | **0.00097** | **-2.51800** | **0.01740** | **0.14690** |
| Intercept | 23.23134 | 2.09617 | 11.08300 | 0.00000 |  | Intercept | 25.01239 | 0.96324 | 25.96700 | <2e-16 |  |
| Precipitation of wettest month (bio13) | -0.01796 | 0.00924 | -1.94300 | 0.06150 | 0.08216 | Precipitation of wettest month (bio13) | **-0.00936** | **0.00425** | **-2.20500** | **0.03530** | **0.11080** |
| Intercept | 17.65659 | 1.04839 | 16.84200 | <2e-16 |  | Intercept | 23.36784 | 0.52477 | 44.53000 | <2e-16 |  |
| Precipitation of driest quarter (bio17) | **0.01794** | **0.00771** | **2.32800** | **0.02680** | **0.12480** | Precipitation of driest quarter (bio17) | -0.00348 | 0.00386 | -0.90100 | 0.37500 | -0.00612 |
| Intercept | 18.78622 | 1.56002 | 12.04200 | 0.00000 |  | Intercept | 23.41087 | 0.72646 | 32.22600 | <2e-16 |  |
| Nitrogen in soil | 0.00123 | 0.00260 | 0.47100 | 0.64100 | -0.02574 | Nitrogen in soil | -0.00074 | 0.00121 | -0.61300 | 0.54500 | -0.02056 |
| Intercept | 16.34719 | 1.91717 | 8.52700 | 0.00000 |  | Intercept | 23.01000 | 0.93920 | 24.49800 | <2e-16 |  |
| Soil organic carbon | 0.00559 | 0.00321 | 1.74200 | 0.09170 | 0.06164 | Soil organic carbon | 0.00003 | 0.00157 | 0.01900 | 0.98500 | 0.00000 |
| Intercept | 13.90745 | 4.31120 | 3.22600 | 0.00303 |  | Intercept | 21.76810 | 2.05539 | 10.59100 | 0.00000 |  |
| Cation exchange capacity | 0.02323 | 0.01787 | 1.30000 | 0.20359 | 0.02175 | Cation exchange capacity | 0.00530 | 0.00852 | 0.62200 | 0.53900 | 0.00000 |
| Intercept | 31.10430 | 7.60110 | 4.09200 | 0.00030 |  | Intercept | 20.15819 | 3.64926 | 5.52400 | 0.00001 |  |
| pH in soil | -0.19970 | 0.12930 | -1.54500 | 0.13292 | 0.04279 | pH in soil | 0.04903 | 0.06208 | 0.79000 | 0.43600 | 0.00000 |
| **Response: Cone length PGLS** |  |  |  |  |  | **Response: DNA content PGLS** |  |  |  |  |  |
| **Estimate** | **Coefficients** | **Std. Error** | ***t-value*** | ***p-value*** | ***λ*** | **Estimate** | **Coefficients** | **Std. Error** | ***t-value*** | ***p-value*** | ***λ*** |
| Intercept | 2.31368 | 0.25106 | 9.21571 | 0.00000 |  | Intercept | 3.30785 | 0.09807 | 33.72792 | 0.00000 |  |
| Precipitation of wettest month (bio13) | -0.00198 | 0.00098 | -2.03014 | 0.05100 | 0.15081 | Annual mean temp. (bio1) | **-0.01143** | **0.00449** | **-2.54817** | **0.01600** | **0.92617** |
|  |  |  |  |  |  | Intercept | 3.13162 | 0.06425 | 48.74470 | 0.00000 |  |
|  |  |  |  |  |  | Min temp. of coldest month (bio6) | -0.00446 | 0.00289 | -1.54248 | 0.13310 | 0.94664 |
|  |  |  |  |  |  | Intercept | 3.18827 | 0.07862 | 40.55368 | 0.00000 |  |
|  |  |  |  |  |  | Annual precipitation (bio12) | -0.00007 | 0.00004 | -1.70248 | 0.09870 | 0.94323 |
|  |  |  |  |  |  | Intercept | 3.17286 | 0.08370 | 37.90712 | 0.00000 |  |
|  |  |  |  |  |  | Precipitation of wettest month (bio13) | -0.00028 | 0.00022 | -1.25841 | 0.21760 | 0.95281 |

**Table S4.** AIC, delta AIC and AICw values for model fit of hard pines characteristics**.**

| **Character** | **Model** | **AIC** | **delta AIC** | **AICw** |
| --- | --- | --- | --- | --- |
| Needles per fascicle | Brownian Motion | 11.2357 | 3.4258 | 0.1462 |
|  | Ornstein-Uhlenbeck | **7.8099** | **0.0000** | **0.8104** |
|  | Early burst | 13.6634 | 5.8535 | 0.0434 |
| Needle length | Brownian Motion | 53.8229 | 15.7889 | 0.0004 |
|  | Ornstein-Uhlenbeck | **38.0339** | **0.0000** | **0.9995** |
|  | Early burst | 56.2506 | 18.2166 | 0.0001 |
| Number of resin canals | Brownian Motion | **12.1779** | **0.0000** | **0.5313** |
|  | Ornstein-Uhlenbeck | 13.2497 | 1.0718 | 0.3109 |
|  | Early burst | 14.6055 | 2.4276 | 0.1578 |
| Cone length | Brownian Motion | **38.2697** | **0.0000** | **0.6077** |
|  | Ornstein-Uhlenbeck | 40.3788 | 2.1091 | 0.2117 |
|  | Early burst | 40.6973 | 2.4276 | 0.1805 |
| Bark thickness | Brownian Motion | **1.2399** | **0.0000** | **0.5392** |
|  | Ornstein-Uhlenbeck | 3.6675 | 2.4276 | 0.1602 |
|  | Early burst | 2.4080 | 1.1681 | 0.3007 |
| DNA content | Brownian Motion | -81.6369 | 6.1367 | 0.0439 |
|  | Ornstein-Uhlenbeck | -79.2093 | 8.5643 | 0.0130 |
|  | Early burst | **-87.7736** | **0.0000** | **0.9431** |
| Annual mean temp. (bio1) | Brownian Motion | **-9.2036** | **0.0000** | **0.4927** |
|  | Ornstein-Uhlenbeck | -8.5808 | 0.6227 | 0.3609 |
|  | Early burst | -6.7759 | 2.4276 | 0.1464 |
| Max. temp. of warmest month (bio5) | Brownian Motion | **-58.2328** | **0.0000** | **0.4481** |
|  | Ornstein-Uhlenbeck | -58.0980 | 0.1349 | 0.4188 |
|  | Early burst | -55.8052 | 2.4276 | 0.1331 |
| Min temp. of coldest month (bio6) | Brownian Motion | **195.2194** | **0.0000** | **0.6068** |
|  | Ornstein-Uhlenbeck | 197.3138 | 2.0944 | 0.2129 |
|  | Early burst | 197.6470 | 2.4276 | 0.1803 |
| Temp. Annual range (bio7) | Brownian Motion | 20.2951 | 26.6316 | 0.0000 |
|  | Ornstein-Uhlenbeck | 22.7227 | 29.0592 | 0.0000 |
|  | Early burst | **-6.3365** | **0.0000** | **1.0000** |

**Table S4.** AIC, delta AIC and AICw values for model fit of hard pines characteristics**.** (continued…)

| **Character** | **Model** | **AIC** | **delta AIC** | **AICw** |
| --- | --- | --- | --- | --- |
| Annual precipitation (bio12) | Brownian Motion | 65.3931 | 17.3867 | 0.0002 |
|  | Ornstein-Uhlenbeck | **48.0064** | **0.0000** | **0.9998** |
|  | Early burst | 67.8208 | 19.8144 | 0.0000 |
| Precipitation of wettest month (bio13) | Brownian Motion | 68.5280 | 15.5729 | 0.0004 |
|  | Ornstein-Uhlenbeck | **52.9551** | **0.0000** | **0.9995** |
|  | Early burst | 70.9557 | 18.0006 | 0.0001 |
| Precipitation of driest quarter (bio17) | Brownian Motion | 73.2513 | 1.5737 | 0.2862 |
|  | Ornstein-Uhlenbeck | 75.6789 | 4.0013 | 0.0850 |
|  | Early burst | **71.6776** | **0.0000** | **0.6287** |
| Nitrogen in soil | Brownian Motion | 53.4170 | 10.3043 | 0.0057 |
|  | Ornstein-Uhlenbeck | **43.1127** | **0.0000** | **0.9925** |
|  | Early burst | 55.8447 | 12.7320 | 0.0017 |
| Soil organic carbon | Brownian Motion | 80.1627 | 36.4477 | 0.0000 |
|  | Ornstein-Uhlenbeck | **43.7151** | **0.0000** | **1.0000** |
|  | Early burst | 82.5904 | 38.8754 | 0.0000 |
| Cation exchange capacity | Brownian Motion | 4.9174 | 18.4951 | 0.0001 |
|  | Ornstein-Uhlenbeck | **-13.5777** | **0.0000** | **0.9999** |
|  | Early burst | 7.3451 | 20.9228 | 0.0000 |
| pH in soil | Brownian Motion | **-61.6077** | **0.0000** | **0.5751** |
|  | Ornstein-Uhlenbeck | -59.9739 | 1.6338 | 0.2541 |
|  | Early burst | -59.1801 | 2.4276 | 0.1708 |

**Table S5.** Comparison among the different variations of OU models employed to test differences among North American, and Mexican and Caribbean pines. OU1: single optimum Ornstein-Uhlenbeck; OUm: Ornstein-Uhlenbeck with different for species of each region; OUmv: Ornstein-Uhlenbeck assuming different state means in sigma square; OUma: Ornstein-Uhlenbeck assuming multiple alpha.

|  |  |  |  |  |  |  |  | Rate |  |  |
| --- | --- | --- | --- | --- | --- | --- | --- | --- | --- | --- |
| Variable | Model | LnL | AIC | AICc | dAIC | Optimum | SE | Alpha | Sigma 2 | Region |
| Needles per fascicle | OU1 | -2.64532 | 11.29064 | 12.11823 | 440.14460 | 1.24024 | 0.81967 | 0.00391 | 0.00599 | North America |
|  |  |  |  |  |  | 1.24024 | 0.81967 | 0.00391 | 0.00599 | Mexico and the Caribbean |
|  | OUM | 14.38078 | -12.76156 | -6.76156 | 421.26480 | 0.98737 | 0.54906 | 0.00315 | 0.00210 | North America |
|  |  |  |  |  |  | 0.00000 | 0.00000 | 0.00315 | 0.00210 | Mexico and the Caribbean |
|  | OUMV | 55.65584 | -85.31168 | -66.15378 | 361.87259 | 1.136791 | 0.200109 | 0.000000 | 0.006375 | North America |
|  |  |  |  |  |  | 0.000000 | 0.000000 | 0.000000 | 0.026747 | Mexico and the Caribbean |
|  | OUMA | **236.59210** | **-447.18430** | **-428.02640** | **0.00000** | 2.000000 | 0.000019 | 1.420710 | 0.000000 | North America |
|  |  |  |  |  |  | 0.000000 | 0.000000 | 0.339913 | 0.000000 | Mexico and the Caribbean |
| Needle length | OU1 | -2.64532 | 11.29065 | 12.11823 | 417.08570 | 1.240237 | 0.819665 | 0.003911 | 0.005994 | North America |
|  |  |  |  |  |  | 1.240237 | 0.819665 | 0.003911 | 0.005994 | Mexico and the Caribbean |
|  | OUM | 36.23922 | -18.47845 | 283.92155 | 688.88902 | 0.005333 | 0.000583 | 0.005333 | 0.000583 | North America |
|  |  |  |  |  |  | 0.005333 | 0.000583 | 0.005333 | 0.000583 | Mexico and the Caribbean |
|  | OUMV | **113.90480** | **-125.80960** | **-404.96750** | **0.00000** | 3.042426 | 0.077845 | 0.055421 | 0.000000 | North America |
|  |  |  |  |  |  | 1.393533 | 0.225138 | 0.055421 | 0.000000 | Mexico and the Caribbean |
|  | OUMA | 40.13788 | 21.72425 | -257.43365 | 147.53382 | 13.184302 | 0.743741 | 0.008843 | 0.000494 | North America |
|  |  |  |  |  |  | 159346.1 | 137632.2 | 0.000000 | 0.000494 | Mexico and the Caribbean |
| Annual precipitation  (bio12) | OU1 | -2.645322 | 11.290645 | 12.118231 | 1259.476543 | 1.24024 | 0.81967 | 0.00391 | 0.00599 | North America |
|  |  |  |  |  |  | 1.24024 | 0.81967 | 0.00391 | 0.00599 | Mexico and the Caribbean |
|  | OUM | **379.4792** | **-684.9583** | **-1247.3583** | **0.00000** | 1.00000 | 0.00000 | 30.57555 | 0.00000 | North America |
|  |  |  |  |  |  | 1.00000 | 0.00000 | 30.57555 | 0.00000 | Mexico and the Caribbean |
|  | OUMV | 368.0427 | -594.0853 | -856.2392 | 391.1191 | 1.00000 | 0.00001 | 15.28819 | 0.00000 | North America |
|  |  |  |  |  |  | 1.00000 | 0.00001 | 15.28819 | 0.00000 | Mexico and the Caribbean |
|  | OUMA | 430.4013 | -718.8026 | -980.9564 | 266.4019 | 3082.864 | 0.06126 | 0.02986 | 0.00000 | North America |
|  |  |  |  |  |  | 1.00000 | 0.00001 | 0.70950 | 0.00000 | Mexico and the Caribbean |

AICw: AIC weight, dAIC: AIC difference between the model and the best model (lowest AIC);

Optimum: Optimum value according to the OU model; dAIC: AIC difference between the model and the best model (lowest AIC)

**Table S5.** Comparison among the different variations of OU models employed to test differences among North American, and Mexican and Caribbean pines. OU1: single optimum Ornstein-Uhlenbeck; OUm: Ornstein-Uhlenbeck with different for species of each region; OUmv: Ornstein-Uhlenbeck assuming different state means in sigma square; OUma: Ornstein-Uhlenbeck assuming multiple alpha. (continued…)

| Variable | Model | LnL | AIC | AICc | dAIC | Optimum | SE | Alpha | Sigma 2 | Region |
| --- | --- | --- | --- | --- | --- | --- | --- | --- | --- | --- |
| Precipitation of wettest  month (bio13) | OU1 | -2.64532 | 11.29065 | 12.11823 | 1666.67935 | 1.24024 | 0.81967 | 0.00391 | 0.00599 | North America |
|  |  |  |  |  |  | 1.24024 | 0.81967 | 0.00391 | 0.00599 | Mexico and the Caribbean |
|  | OUM | 379.47920 | -684.95830 | -1247.35830 | 407.20280 | 1.00000 | 0.00000 | 30.57555 | 0.00000 | North America |
|  |  |  |  |  |  | 2.00000 | 0.00000 | 30.57555 | 0.00000 | Mexico and the Caribbean |
|  | OUMV | 368.04270 | -594.08530 | -856.23920 | 798.32190 | 0.99964 | 0.00001 | 15.28819 | 0.00000 | North America |
|  |  |  |  |  |  | 2.00000 | 0.00001 | 15.28819 | 0.00000 | Mexico and the Caribbean |
|  | OUMA | **767.20360** | **-1392.40730** | **-1654.56110** | **0.00000** | 0.00000 | 0.00000 | 0.00816 | 0.00000 | North America |
|  |  |  |  |  |  | 2.00000 | 137964.9 | 1.99291 | 0.00000 | Mexico and the Caribbean |
| Nitrogen in soil | OU1 | -2.64532 | 11.29065 | 12.11823 | 2954.65303 | 1.24024 | 0.81967 | 0.00391 | 0.00599 | North America |
|  |  |  |  |  |  | 1.24024 | 0.81967 | 0.00391 | 0.00599 | Mexico and the Caribbean |
|  | OUM | 379.47920 | -684.95830 | -1247.35830 | 1695.17650 | 1.00000 | 0.00000 | 30.57555 | 0.00000 | North America |
|  |  |  |  |  |  | 2.00000 | 0.00000 | 30.57555 | 0.00000 | Mexico and the Caribbean |
|  | OUMV | 368.04270 | -594.08530 | -856.23920 | 2086.29560 | 1.00000 | 0.00000 | 30.57555 | 0.00000 | North America |
|  |  |  |  |  |  | 2.00000 | 0.00000 | 30.57555 | 0.00000 | Mexico and the Caribbean |
|  | OUMA | **1411.19000** | **-2680.38100** | **-2942.535** | **0.00000** | 0.00000 | 0.00000 | 3.41603 | 0.00000 | North America |
|  |  |  |  |  |  | 2.00000 | 0.00000 | 12.12256 | 0.00000 | Mexico and the Caribbean |
| Organic Carbon in soil | OU1 | -2.64532 | 11.29064 | 12.11823 | 1665.39252 | 1.24024 | 0.81967 | 0.00391 | 0.00599 | North America |
|  |  |  |  |  |  | 1.24024 | 0.81967 | 0.00391 | 0.00599 | Mexico and the Caribbean |
|  | OUM | 379.47920 | -684.95830 | -1247.35830 | 405.91600 | 1.00000 | 0.00000 | 30.57555 | 0.00000 | North America |
|  |  |  |  |  |  | 1.00000 | 0.00000 | 30.57555 | 0.00000 | Mexico and the Caribbean |
|  | OUMV | 368.04270 | -594.08530 | -856.23920 | 797.03510 | 0.99964 | 0.00001 | 15.28819 | 0.00000 | North America |
|  |  |  |  |  |  | 2.00000 | 0.00001 | 15.28819 | 0.00000 | Mexico and the Caribbean |
|  | OUMA | **766.56020** | **-1391.12040** | **-1653.27430** | **0.00000** | 0.00000 | 0.00000 | 0.00026 | 0.00000 | North America |
|  |  |  |  |  |  | 2.00000 | 0.00000 | 4.46342 | 0.00000 | Mexico and the Caribbean |

AICw: AIC weight, dAIC: AIC difference between the model and the best model (lowest AIC);

Optimum: Optimum value according to the OU model; dAIC: AIC difference between the model and the best model (lowest AIC)

**Table S5.** Comparison among the different variations of OU models employed to test differences among North American, and Mexican and Caribbean pines. OU1: single optimum Ornstein-Uhlenbeck; OUm: Ornstein-Uhlenbeck with different for species of each region; OUmv: Ornstein-Uhlenbeck assuming different state means in sigma square; OUma: Ornstein-Uhlenbeck assuming multiple alpha. (continued…)

| Variable | Model | LnL | AIC | AICc | dAIC | Optimum | SE | Alpha | Sigma 2 | Region |
| --- | --- | --- | --- | --- | --- | --- | --- | --- | --- | --- |
| Cation exchange capacity | OU1 | -2.64532 | 11.29065 | 12.11823 | 1259.476543 | 1.24024 | 0.81967 | 0.00391082 | 0.00599 | North America |
|  |  |  |  |  |  | 1.24024 | 0.81967 | 0.00391 | 0.00599 | Mexico and the Caribbean |
|  | OUM | **379.47920** | **-684.9583** | **-1352.95830** | **0.00000** | 1.00000 | 0.00000 | 30.57555 | 0.00000 | North America |
|  |  |  |  |  |  | 1.00000 | 0.00000 | 30.57555 | 0.00000 | Mexico and the Caribbean |
|  | OUMV | 368.04270 | -594.0853 | -859.16640 | 391.1191 | 1.00000 | 0.00001 | 15.28819 | 0.00000 | North America |
|  |  |  |  |  |  | 1.00000 | 0.00001 | 15.28819 | 0.00000 | Mexico and the Caribbean |
|  | OUMA | 435.5604 | -729.1208 | -761.40500 | 256.0836 | 2934.495 | 0.05695 | 0.03171 | 0.00000 | North America |
|  |  |  |  |  |  | 1.00000 | 0.00001 | 0.71477 | 0.00000 | Mexico and the Caribbean |

AICw: AIC weight, dAIC: AIC difference between the model and the best model (lowest AIC);

Optimum: Optimum value according to the OU model; dAIC: AIC difference between the model and the best model (lowest AIC)

**Table S6.** Results of overlap (*D*) among hard pines species.

| **Comparison (A-B)** | ***D*** | ***P-value A-B*** | ***P-value B-A*** | **Comparison (A-B)** | ***D*** | ***P-value A-B*** | ***P-value B-A*** |
| --- | --- | --- | --- | --- | --- | --- | --- |
| *P. attenuata - P. chihuahuana* | **0.192956** | **0.009901** | **0.009901** | *P. echinata - P. greggii* var. *greggii* | 0.000000 | 1.000000 | 1.000000 |
| *P. attenuata - P. elliottii* | 0.056546 | 0.138614 | 0.099010 | *P. echinata - P. taeda* | 0.437150 | 0.089109 | 0.089109 |
| *P. attenuata - P. herrerae* | 0.123806 | 0.217822 | 0.207921 | *P. elliottii - P. greggii* var. *greggii* | 0.000000 | 1.000000 | 1.000000 |
| *P. attenuata - P. muricata* | 0.034074 | 0.495050 | 0.485149 | *P. elliottii - P. herrerae* | 0.032156 | 0.148515 | 0.148515 |
| *P. attenuata - P. occidentalis* | 0.000000 | 1.000000 | 1.000000 | *P. elliottii - P. leiophylla* | 0.000000 | 0.356436 | 0.366337 |
| *P. attenuata - P. oocarpa* | **0.053921** | 0.069307 | **0.049505** | *P. elliottii - P. muricata* | 0.011685 | 0.504950 | 0.455446 |
| *P. attenuata - P. patula* var. *patula* | **0.006855** | **0.019802** | **0.019802** | *P. elliottii - P. occidentalis* | 0.076987 | 0.227723 | 0.336634 |
| *P. attenuata - P. radiata* var. *radiata* | 0.035829 | 0.346535 | 0.019802 | *P. elliottii - P. oocarpa* | 0.090811 | 0.118812 | 0.089109 |
| *P. attenuata - P. radiata* var. *binata* | 0.011507 | 0.534653 | 0.485149 | *P. elliottii - P. palustris* | 0.558325 | 0.049505 | 0.069307 |
| *P. attenuata - P. taeda* | 0.070969 | 0.059406 | 0.108911 | *P. elliottii - P. patula* var. *patula* | **0.176888** | 0.059406 | **0.029703** |
| *P. chihuahuana - P. elliottii* | 0.000000 | 0.277228 | 0.297030 | *P. elliottii - P. radiata* var. *radiata* | 0.000000 | 1.000000 | 1.000000 |
| *P. chihuahuana - P. taeda* | 0.000000 | 1.000000 | 1.000000 | *P. elliottii - P. tecunumanii* | 0.112939 | 0.445545 | 0.138614 |
| *P. cubensis - P. oocarpa* | **0.147394** | 0.049505 | **0.029703** | *P. glabra - P. oocarpa* | 0.004473 | 0.405941 | 0.366337 |
| *P. cubensis - P. patula* var. *longipedunculata* | **0.025162** | 0.069307 | **0.019802** | *P. greggii* var. *greggii - P. luzmariae* | 0.030773 | 0.207921 | 0.148515 |
| *P. cubensis - P. tecunumanii* | **0.457975** | **0.009901** | **0.009901** | *P. greggii* var. *greggii - P. echinata* | 0.000000 | 1.000000 | 1.000000 |
| *P. echinata - P. elliottii* | 0.039799 | 0.297030 | 0.287129 | *P. greggii* var. *greggii - P. elliottii* | 0.000000 | 1.000000 | 1.000000 |
|  |  |  |  | *P. greggii* var. *greggii - P. occidentalis* | 0.000000 | 1.000000 | 1.000000 |

**Table S6.** Results of overlap (*D*) among hard pines species. (continued…)

| **Comparison (A-B)** | ***D*** | ***P-value A-B*** | ***P-value B-A*** | **Comparison (A-B)** | ***D*** | ***P-value A-B*** | ***P-value B-A*** |
| --- | --- | --- | --- | --- | --- | --- | --- |
| *P. greggii* var. *greggii - P. palustris* | 0.000000 | 1.000000 | 1.000000 | *P. oocarpa - P. tecunumanii* | 0.454023 | 0.069307 | 0.069307 |
| *P. greggii* var. *greggii - P. radiata var. radiata* | 0.139373 | 0.079208 | 0.069307 | *P. teocote - P. oocarpa* | 0.212095 | 0.089109 | 0.059406 |
| *P. greggii* var. *australis - P. patula* var. *longipedunculata* | 0.114860 | 0.108911 | 0.118812 | *P. oocarpa - P. pungens* | 0.000000 | 1.000000 | 1.000000 |
| *P. herrerae - P. rigida* | 0.000000 | 1.000000 | 1.000000 | *P. palustris - P. patula* var. *longipedunculata* | 0.000000 | 1.000000 | 1.000000 |
| *P. leiophylla - P. luzmariae* | **0.366293** | **0.049505** | 0.069307 | *P. palustris - P. radiata* var. *binata* | 0.000000 | 1.000000 | 1.000000 |
| *P. leiophylla - P. palustris* | 0.000000 | 1.000000 | 1.000000 | *P. palustris - P. taeda* | **0.313698** | 0.059406 | **0.039604** |
| *P. leiophylla - P. pringlei* | 0.176483 | 0.079208 | 0.079208 | *P. palustris - P. tecunumanii* | 0.015337 | 0.396040 | 0.425743 |
| *P. leiophylla - P. radiata* var. *radiata* | **0.569972** | **0.009901** | **0.029703** | *P. patula* var. *patula - P. taeda* | 0.009433 | 0.217822 | 0.188119 |
| *P. leiophylla - P. serotina* | 0.029148 | 0.099010 | 0.089109 | *P. patula* var. *longipedunculata - P. radiata* var. *binata* | 0.001495 | 0.415842 | 0.425743 |
| *P. lumholtzii - P. muricata* | **0.508896** | **0.029703** | **0.009901** | *P. patula* var. *longipedunculata - P. serotina* | **0.040700** | **0.049505** | **0.049505** |
| *P. lumholtzii - P. occidentalis* | 0.068946 | 0.059406 | 0.099010 | *P. pungens - P. serotina* | 0.226787 | 0.118812 | 0.148515 |
| *P. lumholtzii - P. palustris* | 0.000000 | 1.000000 | 1.000000 | *P. tecunumanii - P. serotina* | 0.058551 | 0.128713 | 0.207921 |
| *P. lumholtzii - P. radiata* var. *radiata* | **0.467116** | **0.039604** | **0.019802** | *P. tecunumannii - P. radiata* var. *radiata* | 0.022987 | 0.306931 | 0.297030 |
| *P. lumholtzii - P. rigida* | 0.000000 | 1.000000 | 1.000000 | *P. patula* var. *patula - P. serotina* | **0.199549** | **0.009901** | **0.009901** |
| *P. luzmariae - P. patula* var. *longipedunculata* | 0.043495 | 0.247525 | 0.148515 | *P. patula* var. *patula - P. occidentalis* | **0.146167** | **0.039604** | **0.049505** |
| *P. muricata - P. patula* var. *longipedunculata* | 0.079497 | 0.148515 | 0.148515 | *P. greggii* var. *australis - P. cubensis* | **0.042252** | **0.049505** | 0.059406 |
| *P. muricata - P. taeda* | 0.000000 | 1.000000 | 1.000000 | *P. greggii* var. *australis - P. serotina* | 0.017697 | 0.069307 | 0.089109 |
| *P. muricata - P. teocote* | **0.462682** | **0.019802** | **0.009901** | *P. greggii* var. *australis - P. elliottii* | **0.173044** | **0.049505** | **0.039604** |
| *P. muricata - P. oocarpa* | 0.080196 | 0.207921 | 0.188119 | *P. teocote - P. serotina* | **0.034086** | **0.029703** | **0.049505** |
| *P. occidentalis - P. serotina* | 0.017580 | 0.445545 | 0.336634 | *P. teocote - P. radiata* var. *binata* | **0.374321** | **0.009901** | **0.019802** |
| *P. occidentalis - P. tecunumanii* | **0.216049** | **0.029703** | **0.019802** | *P. herrerae - P. muricata* | **0.334102** | **0.029703** | **0.029703** |
| *P. occidentalis - P. teocote* | 0.000000 | 1.000000 | 1.000000 | *P. luzmariae - P. muricata* | **0.287264** | 0.069307 | **0.039604** |
| *P. oocarpa - P. serotina* | 0.029950 | 0.306931 | 0.247525 | *P. luzmariae - P. palustris* | 0.000000 | 1.000000 | 1.000000 |
| *P. oocarpa - P. taeda* | 0.000000 | 1.000000 | 1.000000 |  |  |  |  |

**Table S7.** Values of partial ROC, P-value, variables and centroids used for different ecological niche modelling using ellipsoids in hard pines.

| **Species** | **Partial ROC** | ***P-value*** | **Variable** | **Centroids** |
| --- | --- | --- | --- | --- |
| *P. attenuata* | 1.606384 | 0 | Annual mean temp. (bio1) | 123.00000 |
|  |  |  | Max. temp. of warmest month (bio5) | 292.90909 |
|  |  |  | Mean Temperature of Coldest Quarter (bio11) | 62.27273 |
| *P. radiata* var. *radiata* | 1.939202 | 0 | Max. temp. of warmest month (bio5) | 235.00000 |
|  |  |  | Annual precipitation (bio12) | 618.20000 |
|  |  |  | Precipitation of Wettest Quarter (bio16) | 342.80000 |
| *P. radiata* var. *binata* | 1.975115 | 0 | Max. temp. of warmest month (bio5) | 280.75000 |
|  |  |  | Min Temperature of Coldest Month (bio6) | 75.87500 |
|  |  |  | Mean Temperature of Warmest Quarter (bio10) | 211.75000 |
| *P. muricata* | 1.907407 | 0 | Annual mean temp. (bio1) | 136.52632 |
|  |  |  | Isothermality (bio3) | 58.31579 |
|  |  |  | Temperature seasonality (bio4) | 2922.42105 |
| *P. taeda* | 1.271603 | 0 | Mean diurnal range (bio2) | 127.05950 |
|  |  |  | Temperature annual range (bio7) | 328.53570 |
|  |  |  | Annual precipitation (bio12) | 1269.46430 |
| *P. echinata* | 1.245562 | 0 | Annual mean temp. (bio1) | 154.92265 |
|  |  |  | Mean diurnal range (bio2) | 132.76796 |
|  |  |  | Precipitation of driest month (bio14) | 72.46409 |
| *P. glabra* | 1.841187 | 0 | Annual mean temp. (bio1) | 188.40850 |
|  |  |  | Mean diurnal range (bio2) | 127.91550 |
|  |  |  | Isothermality (bio3) | 42.54930 |
|  |  |  | Max. temp. of warmest month (bio5) | 329.21130 |
|  |  |  | Precipitation of wettest month (bio13) | 173.64790 |
| *P. pungens* | 1.712152 | 0 | Annual mean temp. (bio1) | 107.06711 |
|  |  |  | Mean diurnal range (bio2) | 125.63758 |
|  |  |  | Precipitation seasonality (bio15) | 12.10738 |
| *P. rigida* | 1.392558 | 0 | Annual mean temp. (bio1) | 95.53117 |
|  |  |  | Isothermality (bio3) | 31.94851 |
|  |  |  | Precipitation seasonality (bio15) | 10.82656 |

**Table S7.** Values of partial ROC, P-value, variables and centroids used for different ecological niche modelling using ellipsoids in hard pines. (continued…)

| **Species** | **Partial ROC** | ***P-value*** | **Variable** | **Centroids** |
| --- | --- | --- | --- | --- |
| *P. serotina* | 1.864566 | 0 | Annual mean temp. (bio1) | 184.26316 |
|  |  |  | Mean diurnal range (bio2) | 120.94737 |
|  |  |  | Annual precipitation (bio12) | 1321.10526 |
|  |  |  | Precipitation of wettest month (bio13) | 175.73684 |
|  |  |  | Precipitation of driest month (bio14) | 67.78947 |
| *P. elliottii* | 1.78227 | 0 | Mean diurnal range (bio2) | 116.01690 |
|  |  |  | Max. temp. of warmest month (bio5) | 325.98310 |
|  |  |  | Mean Temperature of Warmest Quarter (bio10) | 269.83050 |
|  |  |  | Precipitation of wettest month (bio13) | 189.18640 |
|  |  |  | Precipitation of driest month (bio14) | 60.93220 |
| *P. palustris* | 1.255344 | 0 | Annual mean temp. (bio1) | 183.67930 |
|  |  |  | Max. temp. of warmest month (bio5) | 326.54310 |
|  |  |  | Annual precipitation (bio12) | 1348.97070 |
| *P. cubensis* | 1.982187 | 0 | Mean diurnal range (bio2) | 96.28571 |
|  |  |  | Temperature seasonality (bio4) | 1614.85714 |
|  |  |  | Precipitation seasonality (bio15) | 48.57143 |
| *P. occidentalis* | 1.97685 | 0 | Temperature seasonality (bio4) | 1286.00000 |
|  |  |  | Precipitation of wettest month (bio13) | 215.20000 |
|  |  |  | Precipitation of driest month (bio14) | 49.80000 |
| *P. patula* var. *patula* | 1.998801 | 0 | Annual mean temp. (bio1) | 132.00000 |
|  |  |  | Mean Temperature of Warmest Quarter (bio10) | 155.80000 |
|  |  |  | Precipitation of driest month (bio14) | 24.60000 |
| *P. patula* var. *longipedunculata* | 1.985506 | 0 | Annual mean temp. (bio1) | 164.60000 |
|  |  |  | Mean Temperature of Warmest Quarter (bio10) | 178.20000 |
|  |  |  | Annual precipitation (bio12) | 1508.00000 |
| *P. greggii* var. *greggii* | 1.9998 | 0 | Annual mean temp. (bio1) | 125.20000 |
|  |  |  | Precipitation of wettest month (bio13) | 85.20000 |
|  |  |  | Precipitation of driest month (bio14) | 15.00000 |

**Table S7.** Values of partial ROC, P-value, variables and centroids used for different ecological niche modelling using ellipsoids in hard pines. (continued…)

| **Species** | **Partial ROC** | ***P-value*** | **Variable** | **Centroids** |
| --- | --- | --- | --- | --- |
| *P. greggii* var. *australis* | 1.981548 | 0 | Mean Temperature of Coldest Quarter (bio11) | 136.66667 |
|  |  |  | Annual precipitation (bio12) | 1373.66667 |
|  |  |  | Precipitation seasonality (bio15) | 79.33333 |
| *P. tecunumanii* | 1.377301 | 0 | Mean diurnal range (bio2) | 117.63158 |
|  |  |  | Annual precipitation (bio12) | 1674.28947 |
|  |  |  | Precipitation of driest month (bio14) | 22.13158 |
| *P. chihuahuana* | 1.786322 | 0 | Annual mean temp. (bio1) | 143.71429 |
|  |  |  | Mean diurnal range (bio2) | 163.33333 |
|  |  |  | Isothermality (bio3) | 52.19048 |
|  |  |  | Mean Temperature of Warmest Quarter (bio10) | 211.95238 |
|  |  |  | Precipitation seasonality (bio15) | 82.00000 |
| *P. leiophylla* | 1.927529 | 0 | Annual mean temp. (bio1) | 144.94872 |
|  |  |  | Mean diurnal range (bio2) | 148.74359 |
|  |  |  | Isothermality (bio3) | 64.97436 |
|  |  |  | Precipitation of driest month (bio14) | 7.41026 |
|  |  |  | Precipitation seasonality (bio15) | 91.71795 |
| *P. herrerae* | 1.929234 | 0 | Isothermality (bio3) | 57.90000 |
|  |  |  | Max. temp. of warmest month (bio5) | 270.20000 |
|  |  |  | Precipitation of Driest Quarter (bio17) | 45.10000 |
| *P. lumholtzii* | 1.897608 | 0 | Annual mean temp. (bio1) | 158.63636 |
|  |  |  | Mean diurnal range (bio2) | 145.04545 |
|  |  |  | Precipitation seasonality (bio15) | 97.90909 |
| *P. teocote* | 1.909857 | 0 | Annual mean temp. (bio1) | 154.11110 |
|  |  |  | Mean diurnal range (bio2) | 139.33330 |
|  |  |  | Annual precipitation (bio12) | 870.88890 |

**Table S7.** Values of partial ROC, P-value, variables and centroids used for different ecological niche modelling using ellipsoids in hard pines. (continued…)

| **Species** | **Partial ROC** | ***P-value*** | **Variable** | **Centroids** |
| --- | --- | --- | --- | --- |
| *P. praetermissa* | 1.998262 | 0 | Mean diurnal range (bio2) | 135.60000 |
|  |  |  | Temperature seasonality (bio4) | 2912.80000 |
|  |  |  | Annual precipitation (bio12) | 919.80000 |
| *P. oocarpa* | 1.767516 | 0 | Annual mean temp. (bio1) | 199.97297 |
|  |  |  | Mean diurnal range (bio2) | 137.72973 |
|  |  |  | Isothermality (bio3) | 67.55405 |
|  |  |  | Annual precipitation (bio12) | 1434.12162 |
|  |  |  | Precipitation seasonality (bio15) | 96.81081 |
| *P. luzmariae* | 1.957625 | 0 | Annual mean temp. (bio1) | 162.00000 |
|  |  |  | Isothermality (bio3) | 61.16667 |
|  |  |  | Annual precipitation (bio12) | 993.58333 |
|  |  |  | Precipitation of driest month (bio14) | 4.91667 |
|  |  |  | Precipitation seasonality (bio15) | 99.41667 |
